# Supplementary material for: Unbiasing fermionic quantum Monte Carlo with a quantum computer
Source: Nature. 2022 Mar 16;603(7901):416–20. doi: 10.1038/s41586-021-04351-z (PMC8930773; doi:10.1038/s41586-021-04351-z)
Supplement: Supplementary file 1 — Supplementary text, equations, tables and references [file 41586_2021_4351_MOESM1_ESM.pdf]

nature portfolio

https://doi.org/10.1038/s41586-021-04351-z

Supplementary information

Unbiasing fermionic quantum Monte Carlo with a quantum computer

In the format provided by the authors and unedited

Supplementary Information for Unbiasing Fermionic Quantum Monte Carlo with a Quantum Computer

William J. Huggins,[1,*] Bryan A. O'Gorman,[2] Nicholas C. Rubin,[1]
David R. Reichman,[3] Ryan Babbush,[1] and Joonho Lee[3,1,†]

[1] *Google Quantum AI, Mountain View, CA, USA*
[2] *Berkeley Quantum Information & Computation Center, University of California, Berkeley, CA, USA*
[3] *Department of Chemistry, Columbia University, New York, NY, USA*

A. TECHNICAL INTRODUCTION

Despite the tremendous advances made in theoretical chemistry and physics over the past several decades, problems with substantial electron correlation, namely effects beyond those treatable at the Hartree-Fock level of theory, still present great challenges to the field.[1–4] Electron correlation effects play a central role in many important situations, ranging from the treatment of transition-metal-containing systems to the description of chemical bond breaking. Reaching so-called "chemical accuracy" (accuracy to within 1 kcal/mol) in such applications is the holy grail of quantum chemistry, and is a goal which no single method can currently reliably and scalably achieve.

Among electronic structure methods, projector quantum Monte Carlo (QMC) has proven to be among the most accurate and scalable. QMC implements imaginary-time evolution of a quantum state with stochastic sampling and can produce unbiased ground state energies when the fermionic sign problem is absent, for example in cases with particle-hole symmetry. Widely used QMC methods include diffusion Monte Carlo (DMC), Greens function Monte Carlo (GFMC), full-configuration interaction QMC (FCIQMC)[5,6] and auxiliary-field QMC (AFQMC) approaches.[7] Generally, chemical systems exhibit a fermionic sign problem and this significantly limits the applicability of QMC to small systems due to exponentially decreasing signal-to-noise ratio.[8] Efficient QMC simulations for sizable systems are possible only with a constraint implemented in conjunction with a trial wavefunction on the imaginary-time trajectories, which at the same time introduces a bias in the final ground state energy estimate. In passing, we note that variational QMC may benefit from some of the techniques proposed in this work, which could be interesting to study further in the future.

The accuracy of QMC simulations is, therefore, wholly determined by the quality of the trial wavefunction. In cases where strong electron correlation is not present, using a simple single Slater determinant trial wavefunction obtained from a mean-field (MF) approach leads to accurate approximate ground state energies from QMC. However, for cases where MF wavefunctions are qualitatively wrong, one must resort to other alternatives. The form of wavefunction must be simple enough to evaluate the projection onto a working QMC basis in an efficient manner. The QMC basis takes the form of real-space points in DMC, occupation vectors in lattice GFMC and FCIQMC, and non-orthogonal Slater determinants in AFQMC. The projection onto the QMC basis often scales exponentially with system size for coupled-cluster states and tensor-product states such as matrix product states. Trial wavefunctions consisting of a linear combination of determinants have been widely used due to the simple evaluation of the projection in this case. However, obtaining an accurate linear combination of determinants scales poorly because the number of important determinants generically scales exponentially with system size. Given these facts, there is a need for a new paradigm that allows for more flexible choices of trial wavefunctions which can lead to more accurate QMC algorithms without losing their scalability.

In this work, we have proposed harnessing the power of quantum computers in performing a hybrid quantum-classical QMC simulation, which we refer to as the QC-QMC algorithm. The key observation that we exploit is that it is possible to perform the QMC basis projection for a wide range of wavefunctions in a potentially more efficient manner on quantum computers than on classical computers. This suggests that one may isolate the specific task of the projection from the QMC algorithm and use quantum computers to perform this task and separately communicate this information to a classical computer to continue the QMC calculation. In principle the required quantity is straightforward to approximate using the Hadamard test.[9] However, because the QMC basis projection needs to be performed thousands of times for a single QMC calculation, for Noisy Intermediate-Scale Quantum (NISQ) devices we propose using shadow tomography to characterize the trial wavefunction and evaluate the projection such that the on-line interaction between the quantum and classical device no longer exists. This enables the exploration of the utility of quantum trial wavefunctions without concern for the challenges of tightly coupling high performance classical computing resources with a NISQ device. We demonstrate the usefulness and noise resilience of this approach by producing accurate experiments through Google's Sycamore processor on prototypical strongly correlated chemical

systems such as H_4 in a minimal basis and a quadruple-zeta basis, as well as bond-breaking of N_2 in a triple-zeta basis. We also studied a minimal unit cell model of diamond within a double zeta basis.

B. REVIEW OF PROJECTOR QUANTUM MONTE CARLO

QMC methods are among the most accurate approximate electronic structure approaches, and they can be systematically improved with the use of increasingly sophisticated trial functions. Here, we summarize the essence of the algorithm and discuss a specific QMC method which works in second-quantized space, namely auxiliary-field quantum Monte Carlo (AFQMC). While we focus on developing a strategy tailored for AFQMC in this work, the general discussion is not limited to AFQMC and should be applicable to QMC in general.

1. Projector quantum Monte Carlo

The essence of any projector QMC methods is that one computes the ground state energy and properties via an imaginary-time propagation

$$|\Psi_0\rangle \propto \lim_{\tau\to\infty} \exp\left(-\tau\hat{H}\right)|\Phi_0\rangle = \lim_{\tau\to\infty}|\Psi(\tau)\rangle, \tag{S1}$$

where τ is the imaginary time, $|\Psi_0\rangle$ is the exact ground state and $|\Phi_0\rangle$ is an initial starting wavefunction satisfying $\langle\Phi_0|\Psi_0\rangle \neq 0$. Without any further modification, this is an exact approach to the computation of the ground state wavefunction. In practice, a deterministic implementation of Eq. (S1) scales exponentially with system size and therefore one resorts to a stochastic realization of Eq. (S1) for scalable simulations. Such a stochastic realization is typically referred to as projector QMC.

Unfortunately, a direct implementation of Eq. (S1) via QMC suffers from the infamous fermionic sign problem.[8] In first quantized QMC methods such as DMC, fermionic antisymmetry is not imposed explicitly. Such approaches require the imposition of the fermionic nodal structure using trial wavefunctions to compute the fermionic ground state. The use of an approximate nodal structure introduces a bias. In second quantized QMC methods the sign problem manifests in a different way. The statistical estimates from a second quantizated QMC method exhibit variances that grow exponentially with system size. Therefore for simulations of large systems no meaningful statistical estimates can be obtained. It is then necessary to impose a constraint in the imaginary-time propagation to deal with the the sign problem and to obtain statistical efficiency. An example of such a constraint is the "phaseless" constraint in AFQMC (see below). While such constraints introduce biases in the final estimates, rendering QMC approaches inherently approximate in practice, different constrained approaches will have relative strengths and weaknesses with respect to accuracy and flexibility.

2. Auxiliary-field quantum Monte Carlo

Auxiliary-field quantum Monte Carlo (AFQMC) is a projector QMC method that works in second-quantized space.[10] Therefore, the sign problem in AFQMC manifests in growing variance in statistical estimates. To impose a constraint in the imaginary-time propagation, it is natural to introduce a trial wavefunction that can be used in the importance sampling as well as the constraint. This results in a wavefunction at imaginary time τ expressed as

$$|\Psi(\tau)\rangle = \sum_i w_i(\tau)\frac{|\phi_i(\tau)\rangle}{\langle\Psi_T|\phi_i(\tau)\rangle} \tag{S2}$$

where $|\phi_i(\tau)\rangle$ is the wavefunction of the i-th walker, $w_i(\tau)$ is the weight of the i-th walker, and $|\Psi_T\rangle$ is some *a priori* chosen trial wavefunction. From Eq. (S2), it is evident that the importance sampling is imposed based on the overlap between the walker wavefunction and the trial wavefunction.

Walker wavefunctions in Eq. (S2) are almost always (on the classical computers) chosen to be single Slater determinants and the action of the imaginary propagation, $\exp\left(-\Delta\tau\hat{H}\right)$, for a small time step $\Delta\tau$ in Eq. (S1) transforms the walkers in such a way that they stay within the single Slater determinant manifold via the Hubbard-Stratonovich transformation. In other words, the Hubbard-Stratonovich transformation turns the many-body imaginary time propagator into single-particle imaginary time propagators coupled to Gaussian random variables called auxiliary fields. This property is essential if the computational cost is to grow only polynomially with system size, and is at

the core of the AFQMC algorithm as well as that of another commonly used unconstrained (and therefore unbiased) projector QMC approach called the determinant QMC method.[11]

While repeatedly applying the imaginary time propagator to the wavefunction, the AFQMC algorithm prescribes a particular way to update the walker weight $w_i(\tau)$ in Eq. (S2). In essence, it is necessary that all weights stay real and positive so that the final energy estimator,

$$E(\tau) = \frac{\langle \Psi_T | \hat{H} | \Psi(\tau) \rangle}{\langle \Psi_T | \Psi(\tau) \rangle} = \frac{\sum_i w_i E^{(i)}(\tau)}{\sum_i w_i}, \tag{S3}$$

has a small variance. Here, $E^{(i)}(\tau)$ is so-called the local energy, which is defined as

$$E^{(i)}(\tau) = \frac{\langle \Psi_T | \hat{H} | \phi_i(\tau) \rangle}{\langle \Psi_T | \phi_i(\tau) \rangle}. \tag{S4}$$

We note that Eq. (S3) is not a variational energy expression and is commonly referred to as the "mixed" energy estimator in QMC. The essence of the constraint is that one updates the i-th walker weight from τ to $\tau + \Delta\tau$ using

$$w_i(\tau + \Delta\tau) = w_i(\tau) \times |S_i(\tau)| \times \max(0, \cos\theta_i(\tau)) \tag{S5}$$

where

$$S_i(\tau) = \frac{\langle \Psi_T | \phi_i(\tau + \Delta\tau) \rangle}{\langle \Psi_T | \phi_i(\tau) \rangle}, \tag{S6}$$

and $\theta_i(\tau)$ is the argument of $S_i(\tau)$. This is in a stark contrast with a typical importance sampling strategy which updates the walker weights using $S_i(\tau)$, which does not guarantee the positivity and reality of the walker weights. If $|\Psi_T\rangle$ is exact, this constraint does not introduce any bias, but simply imposes a specific boundary condition on the imaginary propagation which can be viewed as a "gauge-fixing" of the wavefunction. In practice, one does not have access to the exact $|\Psi_T\rangle$ and therefore can only compute an approximate energy whose accuracy wholly depends on the choice of $|\Psi_T\rangle$. Such a constraint is usually referred to as the "phaseless approximation" in the AFQMC literature.

Currently, classically tractable trial wavefunctions that are commonly used are either single determinant trials or take the form of a linear combination of determinants.[12,13] The former is very scalable (up to 500 electrons or so) but can be often inaccurate, especially for strongly correlated systems, while the latter is limited to a small number of electrons (16 or so) but can produce results that are very accurate even for strongly correlated systems. The choice of the trial wavefunction renders AFQMC limited by the evaluation of Eq. (S3) and Eq. (S6). If the computation of either one of these quantities scales exponentially with system size, the resulting AFQMC calculation will be exponentially expensive.

C. QUANTUM-CLASSICAL HYBRID AUXILIARY-FIELD QMC (QC-AFQMC) ALGORITHMS

In the main text, we presented the general philosophy of the QC-QMC algorithm and here we wish to provide more QC-AFQMC-specific details tailored to the experiments presented in this work.

From the perspective of QMC simulations, the main benefit of using a quantum computer is to expand the range of available trial wavefunctions beyond what is efficient classically. Namely, we seek a class of trial wavefunctions that are inherently more accurate than a single determinant trial while bypassing the difficulty of variational optimization on the quantum computer. Among the set of possible trial functions, we are interested in using wavefunctions for which no known polynomial-scaling classical algorithm exists for the exact evaluation of Eq. (S3) and Eq. (S6). The core idea in the QC-AFQMC algorithm is that one can approximately measure Eq. (S3) and Eq. (S6) on the quantum computer and implement the majority of the imaginary-time evolution classically. Our goal is provide a roadmap for quantum computers to apply polynomial-scaling algorithms for the evaluation of Eq. (S3) and Eq. (S6) up to additive errors and thus ultimately to observe quantum advantage in some systems. This clearly separates subroutines into those that need to be run on quantum computers and those on classical computers.

1. Quantum trial wavefunctions

The specific trial functions of interest in this work are simple variants of so-called coupled-cluster (CC) wavefunctions. In quantum chemistry, CC wavefunctions are among the most accurate many-body wavefunctions.[14] They are

defined by an exponential parametrization,

$$|\Psi\rangle = e^{\hat{T}}|\psi_0\rangle, \tag{S7}$$

where $|\psi_0\rangle$ is a single determinant reference wavefunction and the cluster operator $\hat{T}$ is defined as

$$\hat{T} = \sum_{ai} t_i^a a_a^\dagger a_i + \sum_{ijab} t_{ij}^{ab} a_b^\dagger a_a^\dagger a_j a_i + \cdots . \tag{S8}$$

We use $\{i, j, k, \cdots\}$ to denote occupied orbitals and $\{a, b, c, \cdots\}$ for unoccupied orbitals. $\hat{T}$ can be extended to include single excitations (S), double excitations (D), triple excitations (T) and so on. The resulting CC wavefunction is then systematically improvable by including higher-order excitations. The most widely used version involves up to doubles and is referred to as CC with singles and doubles (CCSD). There is no efficient algorithm for variationally determining the CC amplitudes, $\mathbf{t}$; however, there is an efficient projective way to determine these amplitudes and the energy, although the resulting energy determined by this procedure is not variational. Such non-variationality manifests as a breakdown of conventional CC, although it has been suggested that the underlying wavefunction is still qualitatively correct and the projective energy evaluation is partially responsible for this issue.[15]

Employing CCSD (or higher-order CC wavefunctions) within the AFQMC framework is difficult because the overlap between a CCSD wavefunction and an arbitary Slater determinant cannot be calculated efficiently without approximations. This is true for nearly all non-trivial variants of coupled cluster. Notably, there is currently no known efficient classical algorithm for precisely calculating wavefunction overlaps even for the cases of coupled cluster wevefunctions with a limited set of amplitudes, such as generalized valence bond perfect-pairing (PP).[16,17] In QC-AFQMC, we can efficiently approximate the required overlaps of such wavefunctions by using a quantum computer to prepare a unitary version of CC wavefunctions or approximations to them. By using CC wavefunctions that we can obtain circuit parameters classically, we are able to avoid a costly variational optimization procedure on the quantum device.

The simplified CC wavefunction ansatz that we utilize in this work is the generalized valence bond PP ansatz. This ansatz is defined as

$$|\Psi_{\mathrm{PP}}\rangle = e^{\hat{\kappa}} e^{\hat{T}_{\mathrm{PP}}} |\psi_0\rangle \,, \tag{S9}$$

where the orbital rotation operator is defined as

$$\hat{\kappa} = \sum_{pq}^{N_{\mathrm{orbitals}}} (\kappa_{pq}^{\uparrow} - \kappa_{qp}^{\uparrow}) \hat{a}_{p_\uparrow}^\dagger \hat{a}_{q_\uparrow} + (\kappa_{pq}^{\downarrow} - \kappa_{qp}^{\downarrow}) \hat{a}_{p_\downarrow}^\dagger \hat{a}_{q_\downarrow}, \tag{S10}$$

and the PP cluster operator is

$$\hat{T}_{\mathrm{PP}} = \sum_{i}^{N_{\mathrm{pairs}}} t_i \hat{a}_{i_\uparrow^*}^\dagger \hat{a}_{i_\uparrow} \hat{a}_{i_\downarrow^*}^\dagger \hat{a}_{i_\downarrow}. \tag{S11}$$

In this equation, each i is an occupied orbital and each i^* is the corresponding virtual orbital that is paired with the occupied orbital i. We map the spin-orbitals of this wavefunction to qubits using the Jordan-Wigner transformation. We note that the pair basis in t_i is defined in the rotated orbital basis defined by the orbital rotation operator.

Due to its natural connection with valence bond theory which often provides a more intuitive chemical picture than does molecular orbital theory, the PP wavefunction has played an important role in understanding chemical processes.[16] Despite its exponential scaling when implemented exactly on a classical computer, PP in conjunction with AFQMC has been discussed previously; see Ref. 18. We will explore the scaling of the PP-based approach in classical AFQMC and QC-AFQMC in more in detail below because this wavefunction is used in all of our experimental examples (see Section F).

The PP wavefunction is known to provide insufficient accuracy for the ground state energy in many important examples. This is best illustrated in systems where inter-pair correlation becomes important, such as multiple bond breaking processes.[19] While there exist ways to incorporate inter-pair correlation classically,[20–22] in this work we focus on adding multiple layers of hardware-efficient operators to the PP ansatz. There are two kinds of these additional layers that we have explored:

1. The first class of layers includes only density-density product terms of the form

$$e^{J_{ij}\hat{n}_i\hat{n}_j}. \tag{S12}$$

2. The second class includes only "nearest-neighbor" hopping terms between same spin (σ) pairs

$$e^{Q_{ij}\hat{a}^{\dagger}_{i_\sigma}\hat{a}_{j_\sigma}-Q^*_{ij}\hat{a}^{\dagger}_{j_\sigma}\hat{a}_{i_\sigma}}. \tag{S13}$$

In both cases, the i and j orbitals are physically neighboring in the hardware layout. We alternate multiple layers of each kind and apply these layers to the PP ansatz to improve the overall accuracy. The efficacy of these layers varies with their ordering with the choice of the i,j pairs. Lastly, we also employ a full single particle rotation at the end of the hardware-efficient layers. This last orbital rotation can be applied to 1-body and 2-body Hamiltonian matrix elements classically, so we do not have to implement this part on the quantum computer. We refer this orbital rotation as "offline orbital rotation" as noted in Fig. 2. H_4 was the only example where we went beyond the PP wavefunction. When this type of hardware-efficient layers is used, we no longer have an efficient classical algorithm to optimize the wavefunction parameters. In such cases, one can resort to the variational quantum eigensolver to obtain these parameters. Nevertheless, in the case of H_4, the Hilbert space is small enough (4-orbital) that we still could optimize everything classically.

2. Overlap and Local energy evaluation

As mentioned above, the overlap and local energy evaluations are the key subroutines that involve the quantum trial wavefunctions. One approach to the overlap evaluation is to use the Hadamard test.[9] Using modern methods, one could do this without requiring the state preparation circuit to be controlled by an ancilla qubit.[23–25] However, this approach would require a separate evaluation for each walker at every time step. To avoid a steep prefactor in quantum device run time, we propose the use of the technique known as shadow tomography as discussed in Section D. For now, we will assume that one can make a query to the quantum processor to obtain the overlap between a quantum trial state and an arbitrary Slater determinant efficiently up to additive error of the overlap.

With the ability to measure the overlap between $|\Psi_T\rangle$ and an arbitrary single Slater determinant, $|\phi_i(\tau)\rangle$ we can easily estimate the local energy in Eq. (S4). The evaluation of the denominator is just an overlap quantity and an efficient estimation of the denominator is possible via

$$\langle\Psi_T|\hat{H}|\phi_i(\tau)\rangle = \sum_{pr}\langle\Psi_T|\phi_p^r\rangle\langle\phi_p^r|\hat{H}|\phi_i(\tau)\rangle + \sum_{pqrs}\langle\Psi_T|\phi_{pq}^{rs}\rangle\langle\phi_{pq}^{rs}|\hat{H}|\phi_i(\tau)\rangle, \tag{S14}$$

where $|\phi_p^r\rangle$ and $|\phi_{pq}^{rs}\rangle$ denote single and double excitations from $|\phi_i(\tau)\rangle$, respectively. We only need up to double excitations because our Hamiltonian has up to two-body terms. It is then evident that the ability to estimate $\langle\Psi_T|\phi_p^r\rangle$ and $\langle\Psi_T|\phi_{pq}^{rs}\rangle$ efficiently is sufficient to evaluate the entire local energy because the rest of the terms in Eq. (S14) follow from the simple application of the Slater-Condon rule.[26] The number of overlap queries made to the quantum processor scales as $\mathcal{O}(N^4)$ with N being the system size in this algorithm. Other "mixed" local observables can be computed via similar algorithms.

3. Virtual correlation energy

Obtaining the correlation energy outside the "active" space, where the actual quantum resource is spent, is critical for converging our simulation results to the basis set limit (or the continuum limit). The correlation energy outside the active space will be referred to as "virtual correlation energy". We are limited in terms of the number of qubits on NISQ devices, so a procedure to incorporate correlation energy outside the relatively small active space is essential. To this end, a virtual correlation energy strategy has been proposed within the framework of VQE,[27] but this approach comes with a significant measurement overhead due to the requirement of three- and four-body reduced density matrices within the active space.

In this section, our goal is to show that a similar technique for QC-AFQMC exists where we can obtain the virtual correlation energy without any additional qubits or any measurement overhead. We write our trial wavefunction as

$$|\Psi_T\rangle = |\psi_T\rangle \otimes |\psi_{\rm c}\rangle \otimes |0_{\rm v}\rangle, \tag{S15}$$

where $|\psi_T\rangle$ is the quantum trial wavefunction within the active space, $|\psi_c\rangle$ is a Slater determinant composed of occupied orbitals outside the active space (i.e. frozen core orbitals), and $|0_v\rangle$ is a vacuum state in the space of

unoccupied orbitals outside the active space (i.e., frozen virtual orbitals). We want to compute the overlap between $|\Psi_T\rangle$ and a single Slater determinant $|\phi\rangle$

$$\langle\phi|\Psi_T\rangle = \langle\phi|\left(|\psi_T\rangle \otimes |\psi_c\rangle \otimes |0_v\rangle\right) = \sum_{\substack{x\in\{0,1\}^{N_\mathrm{a}}\\ y\in\{0,1\}^{N_\mathrm{c}}\\ z\in\{0,1\}^{N_\mathrm{v}}}} \langle\phi\,|\,x,y,z\rangle\,\langle x\,|\,\psi_T\rangle\,\langle y\,|\,\psi_c\rangle\,\langle z\,|\,0_v\rangle \tag{S16}$$

$$= \sum_{\substack{x\in\{0,1\}^{N_\mathrm{a}}\\ y\in\{0,1\}^{N_\mathrm{c}}\\ z\in\{0,1\}^{N_\mathrm{v}}}} \phi^*(x,y,z)\psi_T(x)\psi_c(y)\delta_{z,0_v} \tag{S17}$$

$$= \sum_{x\in\{0,1\}^{N_\mathrm{a}}} \left(\sum_{y\in\{0,1\}^{N_\mathrm{c}}} \phi^*(x,y,0_v)\psi_c(y)\right)\psi_T(x), \tag{S18}$$

where $\phi(x,y,z) = \langle x,y,z\,|\,\phi\rangle$, $\psi_T(x) = \langle x\,|\,\psi_T\rangle$, $\psi_c(y) = \langle y|\psi_c\rangle$ N_a is the number of active spin orbitals, and N_c and N_v are the number of occupied and unoccupied spin orbitals outside of the active space, respectively. We are using x, y, z to denote bit strings in the space composed of single particle orbitals used to construct $|\Psi_T\rangle$. Because the tensor $\phi^*(x,y,z)$ represents a Slater determinant, it is a special case of what is known as a matchgate tensor with $N_\mathrm{a}+N_\mathrm{c}+N_\mathrm{v}$ open indices. This is also the case for $\psi_c(y)$ and $\delta_{z,0_v}$ (with N_c and N_v open indices respectively). Thus, their contraction $\left(\sum_{y\in\{0,1\}^{N_\mathrm{c}}}\phi^*(x,y,0_v)\psi_c(y)\right)$ is also a matchgate with N_a open indices and support on states of a fixed Hamming weight (i.e. an unnormalized Slater determinant), and can be formed efficiently by contracting over $N_\mathrm{c}+N_\mathrm{v}$ legs with $|\psi_c\rangle \otimes |0_v\rangle$.[28–30] Let $\tilde{\phi}(x)$ denote the resulting matchgate tensor after normalization and $\left|\tilde{\phi}\right\rangle$ the associated state. Then $\left|\tilde{\phi}\right\rangle$ is a normalized Slater determinant in the same Hilbert space as $|\psi_T\rangle$. Thus, we have

$$\langle\phi|\Psi_T\rangle = \langle\phi|\left(|\psi_T\rangle \otimes |\psi_c\rangle \otimes |0_v\rangle\right) = \text{constant} \times \left\langle\tilde{\phi}\,\middle|\,\psi_T\right\rangle, \tag{S19}$$

where the constant can be efficiently evaluated classically by contracting matchgate states and the evaluation of $\left\langle\tilde{\phi}\,\middle|\,\psi_T\right\rangle$ can now be performed on the quantum computer with only N_a qubits.

For the local energy evaluation in Eq. (S4), we leverage the same technique that we used in Eq. (S14). The numerator of the local energy expression is

$$\langle\phi|\hat{H}|\left(|\psi_T\rangle \otimes |\psi_c\rangle \otimes |0_v\rangle\right) = \sum_{pr}\langle\phi|\hat{H}|\phi_p^r\rangle\langle\phi_p^r|\left(|\psi_T\rangle \otimes |\psi_c\rangle \otimes |0_v\rangle\right) + \sum_{pqrs}\langle\phi|\hat{H}|\phi_{pq}^{rs}\rangle\langle\phi_{pq}^{rs}|\left(|\psi_T\rangle \otimes |\psi_c\rangle \otimes |0_v\rangle\right), \tag{S20}$$

and we only need to focus on the computing the following term:

$$\langle\phi_{pq}^{rs}|\left(|\psi_T\rangle \otimes |\psi_c\rangle \otimes |0_v\rangle\right) = \sum_{\substack{x\in\{0,1\}^{N_\mathrm{a}}\\ y\in\{0,1\}^{N_\mathrm{c}}}} \phi_{pq}^{rs}(x,y,0_v)\psi_T(x)\psi_c(y) = \sum_{x\in\{0,1\}^{N_\mathrm{a}}}\left(\sum_{y\in\{0,1\}^{N_\mathrm{c}}}\phi_{pq}^{rs}(x,y,0_v)\psi_c(y)\right)\psi_T(x). \tag{S21}$$

Then $\left(\sum_{y\in\{0,1\}^{N_\mathrm{c}}}\phi_{pq}^{rs}(x,y,0_v)\psi_c(y)\right)$ is the tensor corresponding to a matchgate state itself (with N_a open indices) and thus can be computed efficiently classically. Since an equation of the form Eq. (S19) also holds for $|\phi_{pq}^{rs}\rangle$, the local energy evaluation can be performed on the quantum computer with only N_a qubits.

4. Other QC-QMC approaches

As mentioned in Section B, our QC-QMC algorithm can be specialized to other projector QMC methods. Here, we provide brief details on other QC-QMC methods, lattice GFMC, FCIQMC, and DMC. In lattice GFMC, walkers are represented by a determinant, $|n\rangle$, in the computational basis. Therefore, the fixed-node constraint utilizes the overlap value, $\langle\Psi_T|n\rangle$, which can be estimated by using the quantum computer. Similar strategies can be explored in fixed-node FCIQMC.[6] In DMC, walkers are represented by a vector in $\mathbb{R}^{3N}$, $|\mathbf{r}\rangle$. The fixed-node constraint in DMC then uses the overlap value, $\langle\Psi_T|\mathbf{r}\rangle = \Psi_T(\mathbf{r})$, which can be obtained from the quantum computer.

D. EXPERIMENTAL IMPLEMENTATION VIA SHADOW TOMOGRAPHY

The basic goal of shadow tomography is to estimate properties of a quantum state without resorting to full state tomography. This task was introduced in Ref. 31 and has been considered in a number of subsequent works.[32–40] In the experiments performed in this work, we make use of these tools to approximate the quantities required to perform AFQMC, Eq. (S3) and Eq. (S6). We focus here on the proposal put forward by Huang et al. in Ref. 32. This version of shadow tomography is experimentally simple to implement and compatible with today's quantum hardware.

As we shall explain, the use of shadow tomography makes our experiment particularly efficient in terms of the number of repetitions required to evaluate the required wavefunction overlaps. This allows us to avoid performing a separate set of experiments (e.g. using the Hadamard test) for each timestep and walker. However, this efficiency comes at a cost; the way in which we extract these overlaps from the experimental measurement record requires an exponentially scaling post-processing step. We note that this difficulty is specific to the particular choice we made to demonstrate QC-QMC using AFQMC rather than some other QMC method. For example, if we were using a quantum computer to provide the constraint for a Green's function Monte Carlo calculation, the walker wavefunctions would be computational basis states and we could make use of shadow tomography without this issue. It is an open question whether a more sophisticated measurement strategy could be equally efficient in terms of the number of measurements required while also avoiding this additional bottleneck for QC-AFQMC. Exploring the use of shadow tomography with random fermionic gaussian circuits, as in Ref. 36, seems like a promising direction to explore for this purpose.

In Supplementary Information D 1, we review the general formalism of shadow tomography as proposed in Ref. 32. We continue in Supplementary Information D 2 by showing how we can use shadow tomography to approximate the wavefunction overlaps required to perform QC-QMC and discussing the scaling in terms of the number of measurement repetitions performed on the quantum device. We explain the challenges associated with the classical post-processing of the experimental record for QC-AFQMC in Supplementary Information D 3. In Supplementary Information D 4 and Supplementary Information D 5, we describe two strategies we adopt for reducing the number of quantum gates required for our experimental implementation. Supplementary Information D 4 deals with compiling the measurements, while Supplementary Information D 5 explains how we make a tradeoff between the number of gates and the number of measurements. Finally, in Supplementary Information D 6, we show that the quantities we ultimately estimate using the quantum device are resilient to noise, particularly noise during the shadow tomography measurement procedure.

1. Review of Shadow Tomography

Let ρ denote some unknown quantum state. We assume that we have access to N copies of ρ. Let $\{O_i\}$ denote a collection of M observables. Our task is to estimate the quantities $\mathrm{tr}(\rho O_i)$ up to some additive error ϵ for each O_i. The key insight of Ref. 32 is that we can accomplish this efficiently in certain circumstances by randomly choosing measurement operators from a tomographically complete set.

To specify a protocol, we choose an ensemble of unitaries $\mathcal{U}$. We then proceed by randomly sampling $U_k \in \mathcal{U}$ and measuring the state $U_k \rho U_k^\dagger$ in the computational basis to obtain the basis state $|b_k\rangle\langle b_k|$. Consider the state $U_k^\dagger |b_k\rangle\langle b_k| U_k$. In expectation, the mapping from ρ to $U_k^\dagger |b_k\rangle\langle b_k| U_k$ defines a quantum channel,

$$\mathcal{M}(\rho) := \mathbb{E}_k\left[U_k^\dagger |b_k\rangle\langle b_k| U_k\right]. \tag{S22}$$

We require that $\mathcal{M}$ be invertible, which is true if and only if the collection of measurement operators defined by drawing $U \in \mathcal{U}$ and measuring in the computational basis is tomographically complete. Assuming that this is true, we can apply $\mathcal{M}^{-1}$ to both sides of Eq. (S22), yielding

$$\begin{aligned}\rho =& \mathcal{M}^{-1}\Big(\mathbb{E}_k\left[U_k^\dagger |b_k\rangle\langle b_k| U_k\right]\Big)\\ =& \mathbb{E}_k\Big[\mathcal{M}^{-1}\big(U_k^\dagger |b_k\rangle\langle b_k| U_k\big)\Big].\end{aligned} \tag{S23}$$

We call the collection $\Big\{\mathcal{M}^{-1}\big(U_k^\dagger |b_k\rangle\langle b_k| U_k\big)\Big\}$ the classical shadow of ρ.

Many choices for the ensemble $\mathcal{U}$ are possible.[32,36,39,40] Formally, the condition that the measurement channel is invertible is sufficient. In practice, it is also desirable to impose the constraint that the classical post-processing involved in making use of the shadow can be done efficiently. In this work, we utilize randomly selected N-qubit Clifford circuits, as well as tensor products of randomly selected Clifford circuits on fewer qubits.

2. Approximating Wavefunction Overlaps with Shadow Tomography

Let $|\Psi_T\rangle$ denote our trial wavefunction. We restrict ourselves to considering $|\Psi_T\rangle$ that represent fermionic wavefunctions with a definite number of particles $\eta > 0$. We focus on states encoded with the Jordan-Wigner transformation, so that the qubit wavefunction for $|\Psi_T\rangle$ is a superposition of computational basis states with Hamming weight η. Let $|\phi\rangle$ denote our walker wavefunction, which is also a superposition of computational basis states with Hamming weight η. In this section, we explain how to approximate the wavefunction overlap $\langle\phi|\Psi_T\rangle$ using shadow tomography.

Our protocol begins by preparing the state $|\tau\rangle\langle\tau|$ on the quantum computer, where $|\tau\rangle = (|0\rangle + |\Psi_T\rangle)/\sqrt{2}$, with $|0\rangle$ denoting the all-zero (vacuum) state. The wavefunction overlap of interest is therefore equal to

$$\langle\phi|\Psi_T\rangle = 2\langle\phi\,|\tau\rangle\langle\tau|\,0\rangle = 2\,\mathrm{Tr}\left[|\tau\rangle\langle\tau|\cdot|0\rangle\langle\phi|\right], \tag{S24}$$

where we used the fact that $\langle\Psi_T\,|\,0\rangle = \langle\phi\,|\,0\rangle = 0$. If we define the observables

$$\begin{aligned} P_+ &= |0\rangle\langle\phi| + |\phi\rangle\langle 0|\,, \\ P_- &= -i(|0\rangle\langle\phi| - |\phi\rangle\langle 0|), \end{aligned} \tag{S25}$$

then we have

$$\mathrm{Re}(\langle\phi|\Psi_T\rangle) = \mathrm{Tr}\left[|\tau\rangle\,\langle\tau|\,P_+\right], \tag{S26}$$

$$\mathrm{Im}(\langle\phi|\Psi_T\rangle) = \mathrm{Tr}\left[|\tau\rangle\,\langle\tau|\,P_-\right], \tag{S27}$$

where $z = \mathrm{Re}(z) + i\,\mathrm{Im}(z)$ for $z \in \mathbb{C}$. Note that $\mathrm{Tr}\left[P_\pm\right] = 0$ and

$$\mathrm{Tr}\left[P_\pm^2\right] = \mathrm{Tr}\left[|\phi\rangle\langle\phi| + |0\rangle\langle 0|\right] = 2. \tag{S28}$$

assuming $|\phi\rangle$ is a normalized wavefunction. While we use these observables to construct our measurement protocol using shadow tomography, we note in passing that they are also related to the alternative approach based on the ancilla-free Hadamard test.[23–25] By preparing $|\tau\rangle$ and diagonalizing $P_\pm$, one can measure $\langle\phi|\Psi_T\rangle$ without using an ancilla to control the state preparation circuits for $|\phi\rangle$ or $|\Psi_T\rangle$.

Let us assume for now that $\mathcal{U}$ is the Clifford group on N qubits. Therefore, we can use the expression for the inverse channel from Ref. 32,

$$\mathcal{M}^{-1}(X) = (2^N + 1)X - \mathbb{I}, \tag{S29}$$

where X is a placeholder variable. Applying this expression to $U_k^\dagger\,|b_k\rangle\langle b_k|\,U_k$ and using Eq. (S26) and Eq. (S27) to construct our single-shot estimate of $\langle\psi|\Phi_T\rangle$, we have

$$\mathrm{Tr}\left[(P_+ + iP_-)\mathcal{M}^{-1}\big(U_k^\dagger\,|b_k\rangle\langle b_k|\,U_k\big)\right] = (2^N + 1)\,\mathrm{Tr}\left[(P_+ + iP_-)U_k^\dagger\,|b_k\rangle\langle b_k|\,U_k\right]. \tag{S30}$$

The full expression for $\langle\phi|\Psi_T\rangle$ then becomes

$$\langle\phi|\Psi_T\rangle = (2^N + 1)\mathbb{E}_k\Big[\mathrm{Tr}\left[(P_+ + iP_-)U_k^\dagger\,|b_k\rangle\langle b_k|\,U_k\right]\Big] = \tag{S31}$$

$$2(2^N + 1)\mathbb{E}_k\Big[\,\langle\phi|\,U_k^\dagger\,|b_k\rangle\langle b_k|\,U_k\,|0\rangle\,\Big]. \tag{S32}$$

Furthermore, because we are expressing $\langle\phi|\Psi_T\rangle$ in terms of the expectation values of the two operators $P_\pm$ with $\mathrm{Tr}\Big[P_\pm^2\Big] = O(1)$, Theorem 1 of Ref. 32 allows us to bound the number of measurement repetitions we require for a target precision. Specifically, when we take the ensemble of random unitaries to be the Clifford group on all N qubits, as we do in this section, this bound scales with the Hilbert-Schmidt norm of the operators of interest. Consider the case where we would like to estimate the overlap of $|\Psi_T\rangle$ with a collection of M different wavefunctions $\{\phi_i\}$. Let $\tilde{c_i}$ denote our estimate of $\langle\phi_i|\Psi_T\rangle$. We specify a desired accuracy in terms of two parameters, ϵ and δ, by demanding that

$$|\tilde{c_i} - \langle\phi_i|\Psi_T\rangle\,| \leq \epsilon \;\;\forall\;\; 1 \leq i \leq M \tag{S33}$$

with probability at least $1-\delta$. Theorem 1 of Ref. 32 implies that shadow tomography using the N-qubit Clifford group allows us to achieve this accuracy using

$$R = \mathcal{O}\big(\frac{\log(M) - \log(\delta)}{\epsilon^2}\big) \tag{S34}$$

repetitions of state preparation and measurement.

3. Classical Post-processing for Wavefunction Overlaps

In the previous section, we described how we can use shadow tomography to estimate overlaps of the form $\langle\phi\,|\,\Psi_T\rangle$ by evaluating the expression in Eq. (S32), $2(2^N+1)\mathbb{E}_k\Big[\langle\phi|\,U_k^\dagger\,|b_k\rangle\langle b_k|\,U_k\,|0\rangle\Big]$, where the U_k are Clifford circuits and b_k are computational basis states. We explained how these estimates can be made using a modest number of experimental repetitions, even for a large collection of different $|\phi_i\rangle$. However, we have not yet described the classical post-processing required to perform this estimation. This section addresses this aspect of our experiment and explains how the approach we took for our implementation of QC-AFQMC in practice involves an exponentially scaling step. We will utilize the fact that overlap between stabilizer states (including basis states) can be efficiently computed classically using the Gottesman-Knill theorem.[41,42] For instance, the terms $\langle b_k|U_k|0\rangle$ can be efficiently calculated for any Clifford circuit U_k. Therefore, we can just focus on computing $\langle\phi|U_k^\dagger|b_k\rangle$ to evaluate the expression in Eq. (S32).

In special cases, this can be computed efficiently. For example, if $|\phi\rangle = \sum_\alpha c_\alpha\,|\phi_\alpha\rangle$ can be written as a linear combination of a polynomial number of stabilizer states $\{|\phi_\alpha\rangle\}_\alpha$, then we can efficiently compute $\langle\phi_\alpha|U_k^\dagger|b_k\rangle$ for each α and sum them together. QMC methods such as Green's function Monte Carlo where the walker wavefunctions are computational basis states are a special case that trivially satisfies this requirement. Even when $|\phi\rangle$ is not exactly sparse, it may be approximately sparse in the computational basis (in the sense of being close to an exactly sparse state). In such a case, provided that we can sample from $|\phi\rangle$ efficiently (which is possible for a Slater determinant), we could construct a sparse approximation to $|\phi\rangle$ (see, e.g., Ref. 43) and use this state to approximate the overlap. In our QC-AFQMC experiments, we expanded $|\phi\rangle$ in this way, except that we performed a sum over all of the computational basis states with the correct symmetries, incurring an exponential overhead. We emphasize, however, that the cost of this post-processing has no effect on the number of quantum samples needed to produce the classical shadow.

For a general wavefunction $|\phi\rangle$, computing $\left\langle\phi\,\middle|\,U_k^\dagger\,\middle|\,b_k\right\rangle$ may be classically intractable. Specifically, when $|\phi\rangle$ is a Slater determinant, as our walkers are, there is no known way to efficiently compute the desired overlap classically. Existing strategies for approximating the overlap between two states can allow us to bypass this exponential scaling if an additive error is acceptable. In general, it is possible to approximate the overlap between two states up to some additive error provided that one can sample from one of the states in the computational basis and query each of them for the amplitudes of particular bitstrings. Techniques of this sort are used in variational Monte Carlo[7] and have also been studied in the context of dequantizing quantum algorithms. In particular, Ref. 44 showed that for normalized states $|\psi\rangle$, $|\phi\rangle$, the random variable $\frac{\langle\phi\,|\,x\rangle}{\langle\psi\,|\,x\rangle}$ with probability $|\langle x\,|\,\psi\rangle|^2$ has mean $\langle\psi\,|\,\phi\rangle$ and constant variance:

$$\langle\psi|\phi\rangle = \sum_x \langle\psi\,|x\rangle\langle x|\,\phi\rangle = \sum_x \frac{\langle\psi|x\rangle}{\langle\phi|x\rangle}|\langle x|\phi\rangle|^2. \tag{S35}$$

This implies an algorithm to calculate $\langle\psi\,|\,\phi\rangle$ to within ϵ additive error with failure probability at most δ using $O(\frac{1}{\epsilon^2}\log\frac{1}{\delta})$ samples from $|\psi\rangle$ and queries to the amplitudes of $|\psi\rangle$ and $|\phi\rangle$. Unfortunately, the prefactor of $2(2^N+1)$ in Eq. (S32) seems to preclude benefiting from a strategy that estimates $\langle\phi|U_k^\dagger|b_k\rangle$ up to an additive error. This is why we chose to compute the overlap using the exponential scaling enumeration of basis states in our QC-AFQMC experiments.

4. Global Stabilizer Measurements

In this section, we outline a strategy for reducing the size of the circuits required to perform shadow tomography. This strategy leverages the fact that we measure in the computational basis immediately after performing a randomly sampled Clifford. Therefore, any permutation of the computational basis states that occurs immediately prior to measurement is unnecessary.

In general, applying a unitary U and then measuring in the computational basis $\left\{|\mathbf{x}\rangle : \mathbf{x}\in\{0,1\}^N\right\}$, as shadow tomography was originally presented, is equivalent to measuring in the rotated basis $\left\{U^\dagger\,|\mathbf{x}\rangle : \mathbf{x}\in\{0,1\}^N\right\}$. For a set of unitaries $\mathcal{U}$, choosing a unitary therefrom uniformly at random and then measuring in the computational basis is equivalent to measuring the positive operator-valued measure (POVM) $\left\{\frac{1}{|\mathcal{U}|}U^\dagger\,|\mathbf{x}\rangle\,\langle\mathbf{x}|\,U : \mathbf{x}\in\{0,1\}^N, U\in\mathcal{U}\right\}$. Note that the $|\mathcal{U}|\,2^N$ measurement operators need not be distinct (e.g., if the unitaries in $\mathcal{U}$ only permute the computational basis states). In particular, when $\mathcal{U}$ is the set of N-qubit Clifford unitaries $\mathcal{C}_N$, each measurement operator $U^\dagger\,|\mathbf{x}\rangle\,\langle\mathbf{x}|\,U$

is a stabilizer state, and the POVM is

$$\left\{ \frac{2^N}{|\text{stab}_N|} |\psi\rangle \langle\psi| : |\psi\rangle \in \text{stab}_N \right\}, \tag{S36}$$

where stab_N is the set of N-qubit stabilizer states. That the weight of the measurement operators is uniform follows from the symmetry of $\mathcal{U}$ (appending any Clifford to each $U \in \mathcal{U}$ leaves the distribution unchanged); that the uniform weight is $2^N / |\text{stab}_N|$ will be explained later. There are $|\mathcal{C}_N| = 2^{N^2+2N} \prod_{i=1}^N (4^i - 1)$ Clifford unitaries[45] and only $2^N \prod_{i=1}^N (2^i + 1) \ll 2^N |\mathcal{C}_N|$ stabilizer states.[42] This suggests that sampling a uniformly random Clifford is unnecessary. We will now construct a smaller set of $2^{-n} |\text{stab}_N|$ unitaries $\tilde{\mathcal{C}}_N$ such that the corresponding POVM is equivalent to that of $\mathcal{C}_N$. Specifically, $\text{stab}_N = \left\{ U^\dagger |\mathbf{x}\rangle : U \in \tilde{\mathcal{C}}_N, \mathbf{x} \in \{0,1\}^N \right\}$.

Let $\mathcal{F}_N$ be the "H-free" (Hadamard-free) group on N qubits, i.e. the group generated by X, CNOT, CZ. The action of any H-free operator can be written as[45]

$$F(\Gamma, \boldsymbol{\gamma}, \Delta, \boldsymbol{\delta}) |\mathbf{x}\rangle = i^{\mathbf{x}^{\mathrm{T}} \Gamma \mathbf{x}} (-1)^{\boldsymbol{\gamma} \cdot \mathbf{x}} |\Delta \mathbf{x} + \boldsymbol{\delta}\rangle, \tag{S37}$$

where Γ is symmetric Boolean matrix; $\boldsymbol{\gamma}, \boldsymbol{\delta} \in \{0,1\}^N$; and Δ is an invertible Boolean matrix. (A Boolean matrix is one whose entries are 0 or 1.) The action of an H-free operator thus is to simply permute the basis states and add some phase. If we are measuring in the computational basis, the phase does not affect the outcome probabilities and the affine change $\mathbf{x} \mapsto \Delta\mathbf{x} + \boldsymbol{\delta}$ is invertible. Therefore measuring a state in the computational basis and applying the transformation $\mathbf{y} \mapsto \Delta^{-1}(\mathbf{y} + \boldsymbol{\delta})$ to the outcome $\mathbf{y}$ is equivalent to applying $F^\dagger$ and then measuring in the computational basis (i.e., measuring in the basis $\left\{ F |\mathbf{x}\rangle : \mathbf{x} \in \{0,1\}^N \right\}$). As shown by Bravyi and Maslov,[45] any Clifford operator can be written in the form $F \cdot H \cdot F'$, where $F, F' \in \mathcal{F}_N$ and H is a layer of single-qubit Hadamards. In shadow tomography, we apply a Clifford $F \cdot H \cdot F'$ and measure in the computational basis. As explained above, however, the second H-free operator F need not actually be applied; its effect can be implemented entirely in classical post-processing. In general, F and F' are not unique. However, Bravyi and Maslov give a canonical form for Clifford operators (by constraining the H-free operators F, F') that allows for uniform sampling. If we start with their canonical form and "push" as much of F' through the Hadamard layer into F, yielding a new form $\tilde{F} \cdot H \cdot \tilde{F}' = F \cdot H \cdot F'$, and neglect the new final H-free operator $\tilde{F}$, we are left with an operator of the form

$$G(I, \Gamma, \Delta) = \prod_{i \in I} H_i P_i^{\Gamma_{i,i}} \prod_{\substack{i \in I \\ j \in I : j \neq i}} \mathrm{CZ}_{i,j}^{\Gamma_{i,j}} \prod_{\substack{i \in I \\ j \notin I : j > i}} \mathrm{CX}_{i,j}^{\Delta_{i,j}}, \tag{S38}$$

where $I \subset [N]$ is a subset of qubit indices, Γ is a Boolean upper-triangular matrix with support only on I, and Δ is Boolean. Applying a Clifford operator and measuring in the computational basis can thus be replaced by applying an operator of the form in Eq. (S38) and measuring in the computational basis. A priori, we would also need to do post-processing to account for the affine transformation effected by the neglected H-free operator, but in fact this is not needed.

5. Partitioned Shadow Tomography

As we discussed in Supplementary Information D 2, shadow tomography using the N-qubit Clifford group can be used to simultaneously estimate M wavefunction overlaps using a number of samples that scales logarithmically in M. However, performing these measurements on a NISQ devices can be challenging because of the required circuit depth. Alternative choices of the ensemble of random unitaries, $\mathcal{U}$, can alleviate this difficulty. In Ref. 32, Huang et al. consider a second choice of $\mathcal{U}$ where the unitaries $U \in \mathcal{U}$ are instead chosen to be tensor products of single-qubit Clifford operators. This choice leads to especially simple circuits. In the worst case, however, it requires a number of measurements scaling exponentially with the locality of the operators to be estimated.

In the experiments performed in this work, we found it useful to interpolate between these two extremes. Specifically, we use an ensemble of random circuits $\mathcal{U}$ consisting of tensor products of random Clifford circuits on $N/2$ qubits. In this section, we explain how the the techniques for overlap estimation we presented in Supplementary Information D 2 can be generalized to this case. Ref. 32 explains how each choice of $\mathcal{U}$ has an associated norm which can be used to bound the variance of the estimators derived from the classical shadow. We do not work out the norm or the associated bounds on the number of measurements for our partitioned shadow tomography here. Instead, we merely note that it performed well in practice and leave this elaboration for a future work.

Recalling and simplifying the expression in Eq. (S30), we have

$$\langle\phi|\Psi_T\rangle = 2\mathbb{E}_k\Big[\,\langle\phi|\,\mathcal{M}^{-1}\big(U_k^\dagger\,|b_k\rangle\!\langle b_k|\,U_k\big)\,|0\rangle\,\Big]. \tag{S39}$$

We can use an expression like the one from Eq. (S29) to apply the inverse channel, but first we need to specify some notation. We take a partitioning of the N qubits into P parts. Let $N_1, N_2, ...N_P$ be the number of qubits in each part of the partition. We consider a shadow tomography protocol that applies a randomly selected N_p-qubit Clifford to each part, $p \in \{1, 2, ...P\}$. Thus, we have

$$U_k = U_k^1 \otimes U_k^2 \otimes \cdots U_k^P. \tag{S40}$$

The inverse of the shadow tomography measurement channel is simply

$$\mathcal{M}^{-1} = \bigotimes_{p=1}^{P} \mathcal{M}_{N_p}^{-1}, \tag{S41}$$

where, as in Eq. (S29),

$$\mathcal{M}_{N_p}^{-1}(X) = (2^{N_p}+1)X - \mathbb{I}_{N_p}. \tag{S42}$$

where X is a placeholder variable.

Now we specialize to the case where $|\phi\rangle$ is a computational basis state, which we denote by $|\beta\rangle$. We could instead take $|\phi\rangle$ to be any state which is separable between the parts of the partition (or a sum of such states), but specializing to computational basis states is sufficient for our purposes. Let $\left|\beta_p\right\rangle$ denote the component of $|\beta\rangle$ associated with the p-th part of the partition. Using this notation, we can evaluate Eq. (S39) to yield

$$\langle\beta|\Psi_T\rangle = 2\mathbb{E}_k\Big[\prod_{p=1}^{P}(2^{N_p}+1)\left\langle\beta_p\right|U_k^{p\dagger}\,|b_k^p\rangle\!\langle b_k^p|\,U_k^p\left|0_p\right\rangle - \left\langle\beta_p|0_p\right\rangle\Big]. \tag{S43}$$

In carrying out our experiments, we specifically chose to use a partition with two parts, one for each of the spin sectors. All of our walker wavefunctions $|\phi\rangle$ were superpositions of basis states with a Hamming weight η overall and a nonzero number of electrons in each spin sector. Therefore, when we used shadow tomography to evaluate the overlap of our walker wavefunctions $|\phi\rangle$ with $|\Psi_T\rangle$ as described in Supplementary Information D 2 and Supplementary Information D 3, $\left\langle\beta_p|0_p\right\rangle = 0$ for the calculations we performed. Because of this, we were able to evaluate the wavefunction overlaps using the expression

$$\langle\phi|\Psi_T\rangle = \sum_i c_i\left\langle\beta^i\middle|\Psi_T\right\rangle = \sum_i c_i 2\mathbb{E}_k\Big[\prod_{p=1}^{P}(2^{N_p}+1)\left\langle\beta_p^i\right|U_k^{p\dagger}\,|b_k^p\rangle\!\langle b_k^p|\,U_k^p\left|0_p\right\rangle\Big], \tag{S44}$$

where the c_i's are the amplitudes of $|\phi\rangle$ in the computational basis, $\{|\beta^i\rangle\}$.

6. Noise Resilience

We show in this section that, in certain circumstances, noise has a negligible impact on the measurement of overlap ratios such as

$$\frac{\langle\phi_1\,|\,\Psi_T\rangle}{\langle\phi_2\,|\,\Psi_T\rangle}, \tag{S45}$$

where $|\Psi_T\rangle$ is some fixed trial wavefunction and $|\phi_1\rangle, |\phi_2\rangle$ are two arbitrary determinants. Recall that the overlap $\langle\phi_i\,|\,\Psi_T\rangle = 2\,\langle\phi_i\,|\,\rho\,|\,0\rangle$, where $\rho = |\tau\rangle\langle\tau| = (|0\rangle + |\Psi_T\rangle)(\langle 0| + \langle\Psi_T|)/2$.

As a warm up, consider a simple noise model: a global depolarizing channel[46]

$$\rho \mapsto \rho' = (1-p)\rho + p\mathbb{I} \tag{S46}$$

applied right before measurement. Then, neglecting the error in estimating the overlaps due to measurement, our estimate of the overlap becomes

$$\frac{2\langle\phi_1\,|\,\rho'\,|\,0\rangle}{2\langle\phi_2\,|\,\rho'\,|\,0\rangle} = \frac{\langle\phi_1\,|\,\rho'\,|\,0\rangle}{\langle\phi_2\,|\,\rho'\,|\,0\rangle} \tag{S47}$$

$$= \frac{(1-p)\langle\phi_1\,|\,\rho\,|\,0\rangle + p\langle\phi_1\,|\,0\rangle}{(1-p)\langle\phi_2\,|\,\rho\,|\,0\rangle + p\langle\phi_2\,|\,0\rangle} \tag{S48}$$

$$= \frac{(1-p)\langle\phi_1\,|\,\rho\,|\,0\rangle}{(1-p)\langle\phi_2\,|\,\rho\,|\,0\rangle} \tag{S49}$$

$$= \frac{\langle\phi_1\,|\,\rho\,|\,0\rangle}{\langle\phi_2\,|\,\rho\,|\,0\rangle}, \tag{S50}$$

where we used the fact that $\langle\phi_i\,|\,0\rangle = 0$. Thus the depolarizing channel has no effect on our estimate.

Now suppose we were to apply the robust shadow tomography procedure of Ref. 33 to determine the overlap ratio in Eq. (S45). We will assume for now that the state ρ is prepared without error and that we have some unknown noise process occurring during the shadow tomography procedure. We focus first on the case where our ensemble of random unitaries ($\mathcal{U}$) is the Clifford group on all N qubits, which we refer to as the global case. First, we would estimate a noise parameter f. Then we would calculate the classical shadow using the inverse channel

$$\mathcal{M}^{-1}(X) = f^{-1}X - \frac{1-f^{-1}}{2^N}\mathbb{I}, \tag{S51}$$

where X is a placeholder variable. Note that, in the absence of noise, we have $f^{-1} = 2^N + 1$ and we recover Eq. (S29). This yields a single-round estimate of the overlap,

$$2\left\langle\phi_i\,\middle|\,\mathcal{M}^{-1}(U_k^\dagger\,|b_k\rangle\langle b_k|\,U_k)\,\middle|\,0\right\rangle = 2f^{-1}\left\langle\phi_i\,\middle|\,U_k^\dagger\,\middle|\,b_k\right\rangle\langle b_k\,|\,U_k\,|\,0\rangle - \frac{1-f^{-1}}{2^N}\operatorname{Tr}\left[U_k^\dagger\,|b_k\rangle\langle b_k|\,U_k\right]\langle\phi_i\,|\,0\rangle \tag{S52}$$

$$= 2f^{-1}\left\langle\phi_i\,\middle|\,U_k^\dagger\,\middle|\,b_k\right\rangle\langle b_k\,|\,U_k\,|\,0\rangle\,. \tag{S53}$$

As above, the factor of f^{-1} drops out when taking ratios. Therefore, when doing shadow tomography (using global Cliffords) to calculate ratios as above, we get robustness *for free*. That is, we can use the true value in the noiseless case $f = (2^N+1)^{-1}$ as in vanilla shadow tomography and the estimates for the ratios are exactly the same as if we had done robust shadow tomography, *without actually doing robust shadow tomography* (i.e., estimating f and using that estimate to obtain the corrected inverse channel). This is true whenever the assumptions of robust shadow tomography hold, i.e., that the noise is gate-independent, time-stationary and Markovian.

For partitioned shadow tomography with two partitions (as described in Supplementary Information D 5), the same conclusion holds. Ref. 33 describes in detail how robust shadow tomography applies to to a random ensemble consisting of a tensor product of single-qubit Clifford operators. We can apply the same logic to the case when we have a tensor product of random $\frac{N}{2}$-qubit Cliffords. This yields an inverse channel,

$$\mathcal{M}^{-1}(\rho) = \Bigg[2^{-n}\left(f_{0,0}^{-1} - f_{0,1}^{-1} - f_{1,0}^{-1} + f_{1,1}^{-1}\right)\mathbb{I}_n \tag{S54a}$$

$$+\, 2^{-n/2}\left(f_{0,1}^{-1} - f_{1,1}^{-1}\right)\left(\mathbb{I}_{n/2}\otimes\operatorname{Tr}_{P_1}[\rho]\right) \tag{S54b}$$

$$+\, 2^{-n/2}\left(f_{1,0}^{-1} - f_{1,1}^{-1}\right)\left(\operatorname{Tr}_{P_2}[\rho]\otimes\mathbb{I}_{n/2}\right) \tag{S54c}$$

$$+\, f_{1,1}\rho\Bigg], \tag{S54d}$$

where $f_{0,0}, f_{0,1}, f_{1,0}, f_{1,1}$ are four four parameters which characterize the impact of the noise. These parameters could be learned from calibration experiments, but, as we will see, this is unnecessary for our purposes.

In our particular case, the two partitions correspond to two spin sectors. We will assume that $|\psi_i\rangle$ has no overlap with any state of the form $|0\rangle\otimes|\psi\rangle$ or $|\psi\rangle\otimes|0\rangle$; in other words, that the state always has at least one particle of each spin. Now again consider a single-round estimate of the overlap $2\left\langle\phi_i\,\middle|\,\mathcal{M}^{-1}(U_k^\dagger\,|b\rangle\langle b|\,U_k)\,\middle|\,0\right\rangle$, where $U_k = U_1\otimes U_2$

and $|b_k\rangle = |b_1\rangle \otimes |b_2\rangle$. There will be four contributions, corresponding to Eq. (S54a)–Eq. (S54d). The first is zero because $\langle \phi_i \,|\, 0\rangle = 0$. The second is proportional to

$$\left\langle \phi_i \,\middle|\, \mathbb{I}_{n/2} \otimes \mathrm{Tr}_{P_1}[U_k^\dagger |b\rangle \langle b| U_k] \,\middle|\, 0 \right\rangle = \left\langle \phi_i \,\middle|\, \mathbb{I}_{n/2} \otimes U_2^\dagger |b_2\rangle \langle b_2| U_2] \,\middle|\, 0 \right\rangle \tag{S55}$$

$$\propto \langle \phi_i| \left(|0\rangle \otimes U_2^\dagger |b_2\rangle \right) = 0. \tag{S56}$$

The third is also zero for the same reason. That leaves just the last term, so that

$$2 \left\langle \phi_i \,\middle|\, \mathcal{M}^{-1}(U_k^\dagger |b\rangle \langle b| U_k) \,\middle|\, 0 \right\rangle = 2^{-n} f_{1,1} \left\langle \phi_i \,\middle|\, U_k^\dagger \,\middle|\, b \right\rangle \langle b \,|\, U_k \,|\, 0\rangle \,. \tag{S57}$$

Therefore, the inverse channel for the noisy implementation of this form of partitioned shadow tomography would simply rescale all of the estimated overlaps by the same noise parameter (when compared with the inverse channel in the absence of noise). This rescaling cancels out when we calculate the overlap ratios and we get robustness automatically whenever the assumptions of robust shadow tomography are satisfied, just as in the global case.

E. COMPUTATIONAL AND EXPERIMENTAL DETAILS AND SUPPORTIVE NUMERICAL RESULTS

We used quantum computing tools provided in Cirq,[47] qsim,[48] and Fermionic Quantum Emulator.[49] For the shadow tomography experiment, we executed each Clifford circuit measurement 1000 times. It took approximately one second of device time to load each circuit and perform the repeated execution and measurement. The loading time is much larger than the time required to perform the measurement and reset, which is itself much larger than the time required to execute the circuit. We expect these timings to improve with future versions of our quantum processors. Furthermore, a larger chip would support parallel execution of multiple experiments.As a result, it took about four hours for H_4 and N_2 and 14 hours for diamond.

All AFQMC calculations presented here were performed with PAUXY[50] and QMCPACK.[51] All integrals are obtained using PySCF[52] and some of the calculations were verified using Q-Chem.[53] Exact energies within a basis were all obtained using a brute-force approach called heat-bath configuration interaction (HCI).[54] For AFQMC, we used more than 1000 walkers in all numerical data presented here to ensure that the population control bias is negligible. $\Delta t = 0.005$ was used for the time step and the resulting time step error was found to be insignificant. When choosing a set of orbitals for the active space, we decided to use orbitals from a brute-force complete active space self-consistent field (CASSCF) calculation. This is not really a necessary component in our method and in the future one may determine those orbitals by performing an active-space self-consistent calculation with some other lower-scaling methods such as orbital-optimized Møller-Plesset perturbation theory.[55] We do not think that the conclusion of this work will be affected by the choice of single particle basis (i.e., orbitals).

In this section, we will provide the raw data of numerical results that were used in the main text. We will use atomic units for the total energies reported in this section.

1. H_4, 8-qubit experiment

We studied a square geometry of H_4 given as (in angstroms)

$$\begin{aligned} \text{H1} &: (0, 0, 0) \\ \text{H2} &: (0, 0, 1.23) \\ \text{H3} &: (1.23, 0, 0) \\ \text{H4} &: (1.23, 0, 1.23). \end{aligned}$$

To compute the atomization energy, one needs an energy of a single hydrogen atom. Since Hartree-Fock is an exact approach for a single electron system (e.g., a hydrogen atom), all correlated methods considered in this work should be exact for this. For a minimal basis (STO-3G), we used -0.46658185 and for a correlation-consistent quadruple zeta basis (cc-pVQZ) we used -0.499945569 for the hydrogen atom energy.

The classical AFQMC calculations were all performed with a spin-unrestricted Hartree-Fock (UHF) trial wavefunction and we also found that the spin-projection technique (which is often employed to improve the AFQMC results)[18] did not provide any improvement to the AFQMC results. We got -1.96655(4) for STO-3G and -2.10910(8) for cc-pVQZ. CCSD(T) (classical "gold standard") was also performed with a UHF reference wavefunction with energies, -1.961308 (STO-3G) and -2.114275 (cc-pVQZ). The exact energy in the cc-pVQZ basis is -2.112166.

We performed both unpartitioned and partitioned shadow tomography four times for STO-3G and twice for cc-pVQZ. To get some sense for the convergence of the shadow tomography experiments as a function of the number of sampled Cliffords, we compute the variational energy of the trial wavefunction via

$$E_{\text{var}} = \frac{\langle \Psi_T | \hat{H} | \Psi_T \rangle}{\langle \Psi_T | \Psi_T \rangle}, \tag{S58}$$

as a function of the number of Cliffords.

$N_{\text{Cliffords}}$	repeat 1	repeat 2	repeat 3	repeat 4
10	-1.800644	-1.764747	-1.813274	-1.658202
16	-1.823041	-1.802192	-1.840494	-1.730591
28	-1.906644	-1.839835	-1.843326	-1.746749
47	-1.925654	-1.888527	-1.860863	-1.809656
80	-1.909567	-1.869456	-1.887139	-1.846339
136	-1.930880	-1.902309	-1.889992	-1.879164
229	-1.944249	-1.921523	-1.903710	-1.890947
387	-1.947362	-1.934682	-1.910477	-1.901883
652	-1.952416	-1.939853	-1.912790	-1.905250
1100	-1.955544	-1.944651	-1.915073	-1.909122
1856	-1.955028	-1.945966	-1.909558	-1.908038
3129	-1.953877	-1.947763	-1.913386	-1.908835
5276	-1.954697	-1.947323	-1.912284	-1.909315
8896	-1.954930	-1.947458	-1.913889	-1.913068
15000	-1.954356	-1.948894	-1.913894	-1.913082

Table S1. **Experimental data of variational energy for H_4 in STO-3G with partitioned tomography**. Variational energy of $|\Psi_T\rangle$ from four independent repeated partitioned shadow tomography experiments with a different set of random Cliffords for H_4, STO-3G (minimal basis). If the experiment was perfect (i.e., no circuit noise), then the variational energy should approach -1.969512.

$N_{\text{Cliffords}}$	repeat 1	repeat 2	repeat 3	repeat 4
10	-1.643633	-1.798261	-1.671065	-1.462214
16	-1.720721	-1.848279	-1.747911	-1.645383
28	-1.816519	-1.911599	-1.786704	-1.737425
47	-1.867034	-1.920776	-1.777655	-1.819957
80	-1.887030	-1.901445	-1.825170	-1.844560
136	-1.924619	-1.930137	-1.845217	-1.858595
229	-1.929421	-1.933710	-1.847781	-1.871717
387	-1.940266	-1.936080	-1.851352	-1.880681
652	-1.936394	-1.937956	-1.860513	-1.878550
1100	-1.935905	-1.936406	-1.875337	-1.881012
1856	-1.938452	-1.938114	-1.877807	-1.884442
3129	-1.939407	-1.939186	-1.880363	-1.887409
5276	-1.936669	-1.939222	-1.882466	-1.890464
8896	-1.937593	-1.938921	-1.872013	-1.888485
15000	-1.938364	-1.939795	-1.871097	-1.887922

Table S2. **Experimental data of variational energy for H_4 in STO-3G with unpartitioned shadow tomography**. Same as Table S1 but for the unpartitioned shadow tomography experiments.

The corresponding variational energies are shown in Table S1 and Table S2 for a minimal basis set (STO-3G) varying the number of Clifford circuits. Using these trial wavefunctions we computed the phaseless AFQMC energies (i.e., QC-AFQMC energies) as shown in Table S5 and Table S6. There is significant variation in the variational energy depending on the number of Cliffords and whether one uses partitioned shadow tomography or not. Nonetheless, the subsequent AFQMC energy is nearly converged with respect to the number of Cliffords at 15000 and run-to-run variation is negligible. We observe essentially the same qualitative results in the case of cc-pVQZ as shown in Table S7 and Table S8.

Aside from the QC-AFQMC energies obtained from experiments, here we provide the QC-AFQMC energies with ideal (i.e., without circuit noise) trial wavefunctions. For STO-3G, we recover the exact ground state energy, -1.969512 since our ideal trial is exact in this basis. For cc-pVQZ, we obtain -2.11247(2).

$N_{\text{Cliffords}}$	repeat 1	repeat 2
10	-1.996118	-1.658351
16	-1.988746	-1.557607
28	-2.009853	-1.873220
47	-2.019875	-1.976545
80	-2.026756	-1.983726
136	-2.034241	-2.005448
229	-2.030444	-2.045285
387	-2.051324	-2.052698
652	-2.053210	-2.056238
1100	-2.059021	-2.054032
1856	-2.059920	-2.053114
3129	-2.057736	-2.053142
5276	-2.060762	-2.054276
8896	-2.060786	-2.053847
15000	-2.059437	-2.054775

Table S3. **Experimental data of variational energy for H_4 in cc-pVQZ with partitioned tomography**.Variational energy of $|\Psi_T\rangle$ from four independent repeated partitioned shadow tomography experiments with a different set of random Cliffords for H_4, cc-pVQZ (a quadruple-zeta basis). If the experiment was perfect (i.e., no circuit noise), then the variational energy should approach -2.069364.

$N_{\text{Cliffords}}$	repeat 1	repeat 2
10	-1.794532	-1.961018
16	-1.864535	-1.963510
28	-1.971853	-2.015256
47	-2.028933	-2.025942
80	-2.022666	-2.029521
136	-2.044745	-2.032204
229	-2.050697	-2.036077
387	-2.055859	-2.038768
652	-2.054068	-2.042764
1100	-2.055576	-2.047633
1856	-2.054740	-2.049588
3129	-2.055636	-2.051308
5276	-2.056442	-2.052641
8896	-2.056741	-2.052579
15000	-2.056641	-2.051843

Table S4. **Experimental data of variational energy for H_4 in cc-pVQZ with unpartitioned tomography**. Same as Table S3 but for the unpartitioned shadow tomography experiments.

2. N_2, 12-qubit experiment

For N_2, we performed only one set of partitioned shadow tomography experiments with a total of 15000 Cliffords because we observed that our final AFQMC energy varies very slightly run-to-run in the case of H_4. We used a correlation-consistent triple-zeta basis, cc-pVTZ.[56] The classical AFQMC calculations done with UHF trial wavefunctions and the spin-projection technique did not change the results discussed here. Similarly, we used UHF reference states for CCSD(T) calculations. Here, we provide the raw data which was used in Fig. 3 (a). Our exact results are obtained from HCI where the second-order perturbation correction was found to be smaller than 0.002 a.u. We believe that these "exact" results are converged with enough precision that these numbers can be used as a benchmark for this system.

3. Diamond, 16-qubit experiment

For diamond, we used the GTH-PADE pseudopotential[57] and the DZVP-GTH basis.[58] Only the Γ-point was considered in the Brillouin zone sampling and the computational unit cell consists of only two carbon atoms. We used spin-restricted HF (RHF) trial wavefunctions for classical AFQMC calculations and CCSD(T) also employed RHF reference states. The "exact" results are obtained from HCI and the second-order perturbation correction was

$N_{\text{Cliffords}}$	repeat 1	repeat 2	repeat 3	repeat 4
10	-1.96943(5)	-1.98295(6)	-1.96873(6)	-1.9724(1)
16	-1.97376(5)	-1.97385(6)	-1.97175(4)	-1.9672(1)
28	-1.97019(3)	-1.97083(4)	-1.97267(4)	-1.97343(8)
47	-1.97033(2)	-1.96931(3)	-1.97261(4)	-1.97400(7)
80	-1.97016(3)	-1.97398(4)	-1.97061(4)	-1.97038(6)
136	-1.97042(2)	-1.97240(4)	-1.97054(4)	-1.96821(5)
229	-1.97046(2)	-1.97090(2)	-1.96931(4)	-1.96844(5)
387	-1.97019(2)	-1.97076(2)	-1.97010(4)	-1.96831(5)
652	-1.97030(2)	-1.97013(2)	-1.96929(4)	-1.96861(4)
1100	-1.96928(2)	-1.96958(2)	-1.96931(4)	-1.96882(5)
1856	-1.96942(2)	-1.96964(1)	-1.96974(4)	-1.96909(5)
3129	-1.96914(2)	-1.96948(2)	-1.96933(4)	-1.96922(4)
5276	-1.96879(2)	-1.96947(2)	-1.96914(4)	-1.96944(5)
8896	-1.96877(2)	-1.96959(2)	-1.96918(4)	-1.96952(4)
15000	-1.96877(2)	-1.96964(2)	-1.96922(4)	-1.96941(4)

Table S5. **Experimental data of QC-AFQMC energy for H_4 in STO-3G with partitioned shadow tomography**. AFQMC energy using $|\Psi_T\rangle$ from four independent repeated partitioned shadow tomography experiments with a different set of random Cliffords for H_4, STO-3G (minimal basis). The exact ground state energy is -1.969512. The numbers in parentheses indicate the statistical error of the AFQMC energy.

$N_{\text{Cliffords}}$	repeat 1	repeat 2	repeat 3	repeat 4
10	-2.0058(1)	-1.97058(9)	-1.9712(1)	-1.9823(2)
16	-1.9907(1)	-1.96982(8)	-1.97094(9)	-1.9869(1)
28	-1.98318(7)	-1.96711(4)	-1.97036(9)	-1.97288(6)
47	-1.97642(5)	-1.96859(3)	-1.9823(1)	-1.97291(6)
80	-1.97430(4)	-1.97010(5)	-1.9833(1)	-1.96990(5)
136	-1.97131(3)	-1.96846(3)	-1.97343(8)	-1.97025(6)
229	-1.97114(2)	-1.96934(3)	-1.97253(8)	-1.96970(6)
387	-1.96995(2)	-1.97006(3)	-1.97059(8)	-1.96981(6)
652	-1.96982(3)	-1.96995(3)	-1.97024(7)	-1.96980(7)
1100	-1.96975(3)	-1.97054(3)	-1.96955(7)	-1.96958(7)
1856	-1.96940(3)	-1.97017(3)	-1.96886(7)	-1.96975(7)
3129	-1.96926(3)	-1.97013(3)	-1.96884(7)	-1.96984(7)
5276	-1.96940(3)	-1.96999(3)	-1.96931(7)	-1.96968(7)
8896	-1.96950(3)	-1.97011(3)	-1.96918(8)	-1.96954(7)
15000	-1.96952(3)	-1.97022(3)	-1.96943(7)	-1.96930(7)

Table S6. **Experimental data of QC-AFQMC energy for H_4 in STO-3G with unpartitioned shadow tomography**. Same as Table S5 but for the unpartitioned shadow tomography experiments.

found to be smaller than 0.0001 a.u. These results should be good as reference data. We took a total of 50000 Clifford samples to perform a set of partitioned shadow tomography experiments at all lattice constants considered. In Table S10, we present the raw data used for Fig. 3 (b).

4. Quantum Circuit Details

In this section we describe the construction of the particular circuits we used in our experiments. In Table S11 and Table S12, we summarize the quantum resource usage in our experiments and other prior works.

The circuits to be applied have two parts: the part that prepares the superposition of the trial wavefunction and the zero state, and the shadow tomography part that implements the measurement operator.

Our trial wave functions are perfect pairing states, followed by some number preserving fermionic gates in the case of the eight qubit experiment. Because the state we want to prepare is

$$|\tau\rangle = \left(|0\rangle + |\Psi_T\rangle\right)/\sqrt{2}, \tag{S59}$$

it is sufficient to prepare

$$\left(|0\rangle + |\mathrm{PP}(\boldsymbol{\theta})\rangle\right)/\sqrt{2}, \tag{S60}$$

$N_{\text{Cliffords}}$	repeat 1	repeat 2
10	-2.10573(9)	-2.1461(3)
16	-2.10766(9)	-2.1214(5)
28	-2.1095(1)	-2.1344(3)
47	-2.1107(2)	-2.1214(1)
80	-2.11063(5)	-2.1313(2)
136	-2.11039(6)	-2.1220(1)
229	-2.11044(6)	-2.11312(5)
387	-2.11120(7)	-2.11141(4)
652	-2.11026(7)	-2.11176(7)
1100	-2.11090(4)	-2.11105(4)
1856	-2.11067(3)	-2.11131(4)
3129	-2.11055(6)	-2.11120(5)
5276	-2.11105(4)	-2.11090(4)
8896	-2.11119(5)	-2.11092(6)
15000	-2.11081(3)	-2.11098(4)

Table S7. **Experimental data of QC-AFQMC energy for H_4 in cc-pVQZ with partitioned shadow tomography**. AFQMC energy using $|\Psi_T\rangle$ from four independent repeated partitioned shadow tomography experiments with a different set of random Cliffords for H_4, cc-pVQZ (a quadruple-zeta basis). The exact ground state energy is -2.11216599. The numbers in parentheses indicate the statistical error of the AFQMC energy.

$N_{\text{Cliffords}}$	repeat 1	repeat 2
10	-2.1188(2)	-2.1070(1)
16	-2.1146(1)	-2.1080(1)
28	-2.10942(9)	-2.11169(9)
47	-2.10951(6)	-2.11108(7)
80	-2.1111(1)	-2.11219(7)
136	-2.11100(4)	-2.11064(6)
229	-2.11105(4)	-2.11218(6)
387	-2.11069(3)	-2.11197(7)
652	-2.11068(4)	-2.11159(8)
1100	-2.11048(4)	-2.11180(5)
1856	-2.1109(1)	-2.11206(6)
3129	-2.11092(6)	-2.11198(5)
5276	-2.11015(3)	-2.11186(5)
8896	-2.11045(3)	-2.11220(5)
15000	-2.11040(4)	-2.11182(5)

Table S8. **Experimental data of QC-AFQMC energy for H_4 in cc-pVQZ with unpartitioned shadow tomography**. Same as Table S7 but for the unpartitioned shadow tomography experiments.

where

$$|\text{PP}(\boldsymbol{\theta})\rangle = \bigotimes_{i=1}^{N/4} |\text{PP}(\theta_i)\rangle \tag{S61}$$

and N is the number of spin orbitals. We do this by creating a state

$$\left(|0\rangle + |1000\rangle^{\otimes N/4}\right)/\sqrt{2} \tag{S62}$$

using a single-qubit Hadamard and a ladder of CNOT and SWAP gates. Then for each set of 4 qubits corresponding to a pair of spatial orbitals we prepare

$$|\text{PP}(\theta)\rangle = \cos(\theta)\,|1100\rangle + \sin(\theta)\,|0011\rangle \propto \text{CNOT}_{1,2}\text{CNOT}_{3,4}\left(i\text{SWAP}_{1,3}\right)^{\theta}|1000\rangle\,, \tag{S63}$$

where the CNOTs and iSWAP gates leave the zero part of the state unchanged. See the portion of Figure 2 (a) labelled "perfect pairing" for a circuit diagram illustrating this step. Figure 2 (a) also shows a circuit diagram of the aforementioned additional number preserving gates used in the eight qubit experiment. The perfect pairing states, as well as the number preserving gates, are discussed from a quantum chemical perspective in Supplementary Information C 1.

R(Å)	Exact	CCSD(T)	Quantum trial (exp.)	Quantum trial (ideal)	AFQMC	QC-AFQMC (exp.)	QC-AFQMC (ideal)
1.000	-109.366398	-109.365383	-109.017231	-109.025925	-109.3672(3)	-109.36697(7)	-109.36685(9)
1.125	-109.399981	-109.398412	-109.043176	-109.053858	-109.4003(3)	-109.40094(7)	-109.4001(1)
1.250	-109.360887	-109.355280	-109.000672	-109.009988	-109.3603(4)	-109.36085(8)	-109.3604(2)
1.500	-109.233325	-109.215012	-108.874636	-108.880510	-109.2342(3)	-109.23109(9)	-109.2309(2)
1.750	-109.132826	-109.110942	-108.808418	-108.810112	-109.1408(2)	-109.13325(8)	-109.1332(1)
2.000	-109.080654	-109.066772	-108.790143	-108.790084	-109.0939(2)	-109.08341(7)	-109.08298(7)
2.250	-109.061147	-109.053758	-108.788486	-108.792041	-109.07392(8)	-109.06177(7)	-109.06293(6)

Table S9. **Raw data for N_2 potential energy surface for seven bond distances (R).** Note that the energy of our quantum trial here is obtained from a single set of experiment which may vary significantly run-to-run. Quantum trial (ideal) indicates the variational energy of the trial wavefunction evaluated exactly assuming that there is no noise in the circuit execution. Similarly, QC-AFQMC (ideal) represents the QC-AFQMC data obtained with the ideal quantum trial wavefunction.

R(Å)	Exact	CCSD(T)	Quantum trial (exp.)	Quantum trial (ideal)	AFQMC	QC-AFQMC (exp.)	QC-AFQMC (ideal)
2.880	-9.545911	-9.546464	-9.121081	-9.426260	-9.5415(1)	-9.54582(5)	-9.54555(4)
3.240	-10.229155	-10.230100	-8.625292	-10.092197	-10.2241(3)	-10.23051(7)	-10.22837(5)
3.600	-10.560477	-10.562229	-10.277938	-10.405326	-10.5525(2)	-10.55861(8)	-10.55864(6)
3.960	-10.700421	-10.703884	-10.368882	-10.528044	-10.6869(2)	-10.6949(1)	-10.6956(1)
4.320	-10.744089	-10.751103	-10.222206	-10.571136	-10.7177(3)	-10.73701(9)	-10.73810 (9)

Table S10. **Raw data for the diamond cold curve for five lattice constants (R).** Note that the energy of our quantum trial here is obtained from a single set of experiment which may vary significantly run-to-run. Note that these energies include the Madelung constant. Quantum trial (ideal) indicates the variational energy of the trial wavefunction evaluated exactly assuming that there is no noise in the circuit execution. Similarly, QC-AFQMC (ideal) represents the QC-AFQMC data obtained with the ideal quantum trial wavefunction.

Now we discuss how to implement the measurement operators. As discussed in Sec. D 4, the measurement operators have the form

$$G(I,\Gamma,\Delta) = \prod_{i\in I} H_i P_i^{\Gamma_{i,i}} \prod_{\substack{i\in I\\ j\in I: j\neq i}} \mathrm{CZ}_{i,j}^{\Gamma_{i,j}} \prod_{\substack{i\in I\\ j\notin I: j>i}} \mathrm{CX}_{i,j}^{\Delta_{i,j}}. \tag{S64}$$

Let $\tilde{\Gamma} = \Gamma + \Delta$. We can rewrite G as

$$G(I,\Gamma,\Delta) = H^{\otimes n} \prod_{i\in I} P_i^{\Gamma_{i,i}} \prod_{i,j} \mathrm{CZ}_{i,j}^{\tilde{\Gamma}_{i,j}} \prod_{i\notin I} H_i, \tag{S65}$$

i.e., a CZ layer sandwiched by two layers of single-qubit gates. Maslov and Roetteler[63] showed that a CZ layer followed by complete reversal of the qubits can be implemented using a circuit of $2n+2$ CNOT layers (plus intervening layers of single qubit powers of P). Because the CZ layer in the circuit for G is followed only by single-qubit gates and measurement in the computational basis, the reversal of qubits can be easily undone in post-processing. Thus the shadow tomography circuits have a 2-qubit gate depth of at most $2n+2$. This is a significant improvement over using the full Clifford group for shadow tomography; the best known circuit for a general Clifford has 2-qubit depth $9n$.[45] Furthermore, the CZ circuits have the additional properties that they contain only four unique CNOT layers and that they act only along a line, which are advantageous for calibration and qubit mapping, respectively.

F. OUTLOOK ON POTENTIAL QUANTUM ADVANTAGE

In the typical electronic structure context, quantum advantage is focused on the approximation of the ground state energy. In this outlook, we consider the potential for quantum advantage in this general sense, as well as for

Experiment	# Qubits	# CZ Gates (State Prep)	# CZ Gates (Total)	Circuit Depth
Hydrogen (Partitioned)	8	36	66	52
Hydrogen (Unpartitioned)	8	36	99	67
Nitrogen	12	22	92	53
Diamond	16	34	160	65

Table S11. **Resource counts for the QC-AFQMC experiments realized in this work.**

Experiment	Reference	# Qubits	# 2q Gates
BeH_2	[59]	6	5 (U_{ENT})
H_2O	[60]	5	6 ($XX(\theta)$)
Hydrogen	[61]	12	72 ($\sqrt{i\text{SWAP}}$)
Diazene	[61]	10	50 ($\sqrt{i\text{SWAP}}$)
Hubbard, interacting (8-site)	[62]	16	608 ($\sqrt{i\text{SWAP}}$)
Hubbard, non-interacting (8-site)	[62]	16	1568 ($\sqrt{i\text{SWAP}}$)

Table S12. **Resource estimates from prior fermionic simulations using gate model quantum computers on more than four qubits.** For the two Hubbard model experiments we distinguish between dynamics simulated for an interacting versus a non-interacting model. $N = 8$ indicates an eight site linear lattice with open boundary conditions. U_{ENT} is a nearest-neighbor cross-resonance style gate and $XX(\theta)$ is a $\exp(-i\theta\sigma_x^i\sigma_x^j/2)$. As far as we are aware, these are the largest simulations using a gate-model quantum computer targeting fermionic ground states or dynamics.

the specific quantum subroutine used in our QC-AFQMC algorithm, namely the overlap evaluation. We explain our understanding here of the current computational scaling and limits of our proposed approach for the overlap evaluation and the path towards the first "practical" quantum advantage.

System size scaling. In general, we expect the overlap between $\langle\Psi_T|\phi\rangle$ to approach zero exponentially quickly as the system size increases. For example, the typical overlap value of the walker wavefunction with a simple trial wavefunction can be as small as 10^{-5} for 16 atoms, 10^{-16} for 54 atoms, and 10^{-38} for 128 atoms under periodic boundary conditions.[64] These examples suggest that the system size scaling consideration is not just an asymptotic consideration but is practically relevant for system sizes that one may wish to study in the near future. Performing AFQMC requires evaluating these overlaps to a fixed relative precision. Therefore, as the system size increases towards the thermodynamic limit, we would expect that QC-AFQMC formally requires exponentially more measurements to maintain the relative precision.

In order to address the challenges due to this scaling, QC-AFQMC might need to be developed beyond the formulation used in our experiment. For example, using more sophisticated wavefunction forms for $|\phi\rangle$ than a single Slater determinant could allow one to maintain good overlap between $|\Psi_T\rangle$ and $|\phi\rangle$. Again, as long as $|\phi\rangle$ can be prepared efficiently on a quantum computer, one can efficiently estimate the overlap $\langle\Psi_T|\phi\rangle$ to fixed additive error using the Hadamard test. This allows us to efficiently work with both trial and walker wavefunctions that would require an exponentially large multideterminent expansion to accurately represent with a classical computer. In some cases, these overlaps might still be too small as a consequence of the QMA-Hardness of the electronic structure problem.[65] However, the onset of this sort of exponential scaling would also render intractable other quantum computing algorithms such as energy estimation via quantum phase estimation.[66] The reason for this is because such approaches have a cost that is inversely proportional to the overlap between the target eigenstate of interest and the initial state. Thus, if one can make quantum phase estimation efficient by preparing a suitable initial state, we are optimistic that one can use parameterized versions of those states as the initial $|\phi\rangle$ in QC-QMC in order to avoid the problem of vanishing overlaps. We note that VQE is also expected to face similar difficulties in the worst case since no polynomial scaling circuit ansatz is able to prepare ground states of the most challenging instances of the electronic structure problem to target precision in the worst case.

Alternatively, one could pursue strategies for controlling the sign problem which do not require computing the global wavefunction overlaps to a high precision directly. Classically, the exponential decay of these overlap values with respect to system size for single Slater determinant walkers is numerically well handled by computing the log of the overlap value directly and working only with the overlap ratio when performing the AFQMC calculations. While that particular strategy seems difficult to implement on a quantum computer, it seems reasonable that one could leverage the finite correlation length of physical systems to avoid the need for an exponentially growing number of measurements. More specifically, our virtual correlation technique allows for choosing a relatively small physical space to treat with the quantum processor while computing the correlation energy in a much larger space. The use of such a small physical space (known as an active space in quantum chemistry) can be rigorously justified for systems with a finite correlation length. The typical wavefunction overlaps under this approach would therefore be (at worst) exponentially small in a quantity related to the correlation length rather than the size of the system. Furthermore, it is often possible to keep the physical space small by identifying a reduced set of physically relevant degrees of freedom. In practice, the combination of these facts will help us maintain overlaps much larger than we would expect in the most general cases.

Quantum advantage in the overlap estimation. A related but independently interesting question is whether there is a potential for quantum advantage with regards to the specific task of estimating the overlap up to an additive error between some quantum state and an arbitrary walker wavefunction (a single Slater determinant in our particular experiments). Although the use of shadow tomography is guaranteed to be efficient for this task in terms of the number

of measurements, the classical post-processing used in our shadow tomography experiments was performed with an exponential overhead incurred by enumerating all possible determinants in the Hilbert space (see Section D 2 and Section D 3). One open question raised by our work is whether there is a way to remove this exponential overhead in the classical post-processing of shadow tomography for QC-AFQMC, possibly by using a different ensemble of random unitaries. Building on Ref. 36's fermionic shadow tomography seems promising in this regard. Even if the answer is no, one does not need to use shadow tomography; using the Hadamard test, one can obtain the overlaps up to additive error efficiently without any problematic classical post-processing. In fact, the only post-processing required in this case is to compute a simple average over $+1$ and -1-valued measurement outcomes, regardless of the complexity of the states involved. Thus, in general, one can estimate these overlaps up to an additive error in a fashion that is entirely efficient. One could also pursue a version of QC-QMC that uses shadow tomography while avoiding this obstacle by using walkers that are particularly well suited for use with shadow tomography, e.g., composed of a linear combination of stabilizer states (states generated by Clifford circuits). The Green's function Monte Carlo method is one example of this (as the walker wavefunctions are computational basis states).

We employed the perfect pairing (PP) wavefunction as a workhorse in all our experiments. While to the best of our knowledge there is no efficient classical algorithm that can compute the overlap between a PP state and an arbitrary single Slater determinant *exactly*, there is an efficient classical algorithm (see Section D 3) that can approximate this quantity up to some additive error. Therefore, we can assert that there is no quantum advantage in using PP trial wavefunctions in QC-AFQMC. On the other hand, more complex states such as the one used in our H_4 experiment (i.e., PP state with hardware efficient layers), other hardware-efficient wavefunctions, some variants of the unitary coupled-cluster (UCC) wavefunction (see Section C 1), wavefunctions constructed by adiabatic state preparation, and the two-dimensional multiscale entanglement renormalization (2D-MERA) wavefunction may be good candidates for seeking a quantum advantage in the estimation of overlaps. This is due to the fact that no known classical algorithms (including the one described in Section D 3) efficiently yield the overlap of these wavefunctions (up to an additive error) with an arbitrary Slater determinant, or indeed, a computational basis state. Overlaps between all these states and a single Slater determinant can be approximated efficiently up to additive error on the quantum computer using the Hadamard test. Overlaps of these states with stabilizer states (including computational basis states) can be approximated efficiently using existing shadow tomography techniques.

Quantum advantage in the ground state energy computation. When the number of electrons that we consider is not too large, it is possible to assume that the measurement overhead due to the vanishing overlap may not be a practical concern. With our virtual correlation technique, we can maintain a good overlap value within the active space while producing accurate energies overall. Given this, we are optimistic about routes to achieve quantum advantage in fermionic ground state simulation through the QC-AFQMC algorithm. The aforementioned complex quantum states such as hardware efficient ansatze, UCC and 2D-MERA can be good candidates for trial wavefunctions although the relevance of 2D-MERA for chemistry simulations is yet to be seen. An important consideration here is how one actually obtains wavefunction parameters of those complex quantum states. One may optimize them using the variational quantum algorithm or one may take states that can be efficiently optimized classically. For the latter case, it seems likely that approximating the overlap between these states and an arbitrary Slater determinant up to additive error is difficult despite the fact that some of them can be optimized efficiently using classical algorithms. Our prospects for observing a quantum advantage with these sophisticated wavefunctions are further bolstered by the recent observation that improved trial wavefunctions can also ameliorate the sign problem in free projection QMC calculations, where the constraints used to control the sign problem are either removed after some initial propagation time, or not employed at all.[67,68]

We hope to observe quantum advantage either in the overlap estimation or in the ground state energy computation using QC-AFQMC or other variants of QC-QMC and believe that continued advancement along this direction will lead us to one of the first realizations of practical quantum advantage in NISQ fermionic simulations.

---

[*] These two authors contributed equally; corresponding author: whuggins@google.com
[†] These two authors contributed equally; corresponding author: linusjoonho@gmail.com
[1] Richard A. Friesner, "Ab initio quantum chemistry: Methodology and applications," Proc. Natl. Acad. Sci. U.S.A. **102**, 6648–6653 (2005).
[2] Trygve Helgaker, Wim Klopper, and David P. Tew, "Quantitative quantum chemistry," Mol. Phys. **106**, 2107–2143 (2008).
[3] Yudong Cao, Jonathan Romero, Jonathan P. Olson, Matthias Degroote, Peter D. Johnson, Mária Kieferová, Ian D. Kivlichan, Tim Menke, Borja Peropadre, Nicolas P. D. Sawaya, Sukin Sim, Libor Veis, and Alán Aspuru-Guzik, "Quantum Chemistry in the Age of Quantum Computing," Chem. Rev. **119**, 10856–10915 (2019).
[4] Bela Bauer, Sergey Bravyi, Mario Motta, and Garnet Kin-Lic Chan, "Quantum Algorithms for Quantum Chemistry and Quantum Materials Science," Chem. Rev. **120**, 12685–12717 (2020).

[5] George H. Booth, Alex J. W. Thom, and Ali Alavi, "Fermion Monte Carlo without fixed nodes: A game of life, death, and annihilation in Slater determinant space," J. Chem. Phys. **131**, 054106 (2009).
[6] Nick S. Blunt, "Fixed- and Partial-Node Approximations in Slater Determinant Space for Molecules," J. Chem. Theory Comput. **2021** (2021), 10.1021/acs.jctc.1c00500.
[7] Federico Becca and Sandro Sorella, *Quantum Monte Carlo Approaches for Correlated Systems* (Cambridge University Press, Cambridge, England, UK, 2017).
[8] Matthias Troyer and Uwe-Jens Wiese, "Computational Complexity and Fundamental Limitations to Fermionic Quantum Monte Carlo Simulations," Phys. Rev. Lett. **94**, 170201 (2005).
[9] A Yu. Kitaev, "Quantum measurements and the abelian stabilizer problem," (1995), arXiv:quant-ph/9511026 [quant-ph].
[10] Mario Motta and Shiwei Zhang, "Ab initio computations of molecular systems by the auxiliary-field quantum monte carlo method," WIREs Comput. Mol. Sci. **8**, e1364 (2018).
[11] R. Blankenbecler, D. J. Scalapino, and R. L. Sugar, "Monte Carlo calculations of coupled boson-fermion systems. I," Phys. Rev. D **24**, 2278–2286 (1981).
[12] Joonho Lee, Miguel A. Morales, and Fionn D. Malone, "A phaseless auxiliary-field quantum Monte Carlo perspective on the uniform electron gas at finite temperatures: Issues, observations, and benchmark study," J. Chem. Phys. **154**, 064109 (2021).
[13] Wirawan Purwanto, Shiwei Zhang, and Henry Krakauer, "An auxiliary-field quantum monte carlo study of the chromium dimer," J. Chem. Phys. **142**, 064302 (2015).
[14] Rodney J. Bartlett and Monika Musiał, "Coupled-cluster theory in quantum chemistry," Rev. Mod. Phys. **79**, 291 (2007).
[15] Troy Van Voorhis and Martin Head-Gordon, "Benchmark variational coupled cluster doubles results," J. Chem. Phys. **113**, 8873–8879 (2000).
[16] William A. Goddard, Thom H. Dunning, William J. Hunt, and P. Jeffrey Hay, "Generalized valence bond description of bonding in low-lying states of molecules," Acc. Chem. Res. **6**, 368–376 (1973).
[17] John Cullen, "Generalized valence bond solutions from a constrained coupled cluster method," Chem. Phys. **202**, 217–229 (1996).
[18] Wirawan Purwanto, WA Al-Saidi, Henry Krakauer, and Shiwei Zhang, "Eliminating spin contamination in auxiliary-field quantum monte carlo: Realistic potential energy curve of F_2," J. Chem. Phys. **128**, 114309 (2008).
[19] David W. Small and Martin Head-Gordon, "Post-modern valence bond theory for strongly correlated electron spins," Phys. Chem. Chem. Phys. **13**, 19285–19297 (2011).
[20] Troy Van Voorhis and Martin Head-Gordon, "The imperfect pairing approximation," Chem. Phys. Lett. **317**, 575–580 (2000).
[21] David W. Small, Keith V. Lawler, and Martin Head-Gordon, "Coupled Cluster Valence Bond Method: Efficient Computer Implementation and Application to Multiple Bond Dissociations and Strong Correlations in the Acenes," J. Chem. Theory Comput. **10**, 2027–2040 (2014).
[22] Joonho Lee, David W. Small, and Martin Head-Gordon, "Open-shell coupled-cluster valence-bond theory augmented with an independent amplitude approximation for three-pair correlations: Application to a model oxygen-evolving complex and single molecular magnet," J. Chem. Phys. **149**, 244121 (2018).
[23] William J. Huggins, Joonho Lee, Unpil Baek, Bryan O'Gorman, and K. Birgitta Whaley, "A non-orthogonal variational quantum eigensolver," New J. Phys. **22**, 073009 (2020).
[24] Sirui Lu, Mari Carmen Bañuls, and J Ignacio Cirac, "Algorithms for quantum simulation at finite energies," (2020), arXiv:2006.03032 [quant-ph].
[25] A. E. Russo, K. M. Rudinger, B. C. A. Morrison, and A. D. Baczewski, "Evaluating Energy Differences on a Quantum Computer with Robust Phase Estimation," Phys. Rev. Lett. **126**, 210501 (2021).
[26] Attila Szabo and Neil S. Ostlund, *Modern Quantum Chemistry: Introduction to Advanced Electronic Structure Theory* (Courier Corporation, 1996).
[27] T. Takeshita, N.C. Rubin, Z. Jiang, E. Lee, R. Babbush, and J.R. McClean, "Increasing the Representation Accuracy of Quantum Simulations of Chemistry without Extra Quantum Resources," Physical Review X **10** (2020).
[28] Sergey Bravyi, "Contraction of matchgate tensor networks on non-planar graphs," (2008), arXiv:0801.2989 [quant-ph].
[29] M. Hebenstreit, R. Jozsa, B. Kraus, S. Strelchuk, and M. Yoganathan, "All pure fermionic non-gaussian states are magic states for matchgate computations," Physical Review Letters **123** (2019), 10.1103/physrevlett.123.080503.
[30] David P. DiVincenzo and Barbara M. Terhal, "Fermionic linear optics revisited," Foundations of Physics **35**, 1967–1984 (2005).
[31] Scott Aaronson, "Shadow tomography of quantum states," SIAM J. Comput. **49**, STOC18–368–STOC18–394 (2020).
[32] Hsin-Yuan Huang, Richard Kueng, and John Preskill, "Predicting many properties of a quantum system from very few measurements," (2020), arXiv:2002.08953 [quant-ph].
[33] Senrui Chen, Wenjun Yu, Pei Zeng, and Steven T Flammia, "Robust shadow estimation," (2020), arXiv:2011.09636 [quant-ph].
[34] G I Struchalin, Ya A Zagorovskii, E V Kovlakov, S S Straupe, and S P Kulik, "Experimental estimation of quantum state properties from classical shadows," (2020), 10.1038/s41567-020-0932-7, arXiv:2008.05234 [quant-ph].
[35] Dax Enshan Koh and Sabee Grewal, "Classical shadows with noise," (2020), arXiv:2011.11580 [quant-ph].

[36] Andrew Zhao, Nicholas C Rubin, and Akimasa Miyake, "Fermionic partial tomography via classical shadows," (2020), arXiv:2010.16094 [quant-ph].

[37] Dorit Aharonov, Jordan Cotler, and Xiao-Liang Qi, "Quantum algorithmic measurement," (2021), arXiv:2101.04634 [quant-ph].

[38] Hsin-Yuan Huang, Richard Kueng, and John Preskill, "Efficient estimation of pauli observables by derandomization," (2021), arXiv:2103.07510 [quant-ph].

[39] Charles Hadfield, "Adaptive pauli shadows for energy estimation," (2021), arXiv:2105.12207 [quant-ph].

[40] Hong-Ye Hu and Yi-Zhuang You, "Hamiltonian-Driven shadow tomography of quantum states," (2021), arXiv:2102.10132 [quant-ph].

[41] Daniel Gottesman, "Class of quantum error-correcting codes saturating the quantum hamming bound," Phys. Rev. A **54**, 1862–1868 (1996).

[42] Scott Aaronson and Daniel Gottesman, "Improved simulation of stabilizer circuits," (2004), arXiv:quant-ph/0406196 [quant-ph].

[43] Martin Schwarz and Maarten Van den Nest, "Simulating quantum circuits with sparse output distributions," (2013), arXiv:1310.6749 [quant-ph].

[44] Ewin Tang, "A quantum-inspired classical algorithm for recommendation systems," Proceedings of the 51st Annual ACM SIGACT Symposium on Theory of Computing (2019), 10.1145/3313276.3316310.

[45] Sergey Bravyi and Dmitri Maslov, "Hadamard-free circuits expose the structure of the clifford group," (2020), arXiv:2003.09412 [quant-ph].

[46] Michael A. Nielsen and Isaac L. Chuang, *Quantum Computation and Quantum Information: 10th Anniversary Edition* (Cambridge University Press, Cambridge, England, UK, 2010).

[47] Cirq Developers, "Cirq (2021)," See full list of authors on Github: https://github.com/quantumlib/Cirq/graphs/contributors .

[48] Quantum AI team and collaborators, "qsim," (2020).

[49] Nicholas C Rubin, Toru Shiozaki, Kyle Throssell, Garnet Kin-Lic Chan, and Ryan Babbush, "The fermionic quantum emulator," arXiv preprint arXiv:2104.13944 (2021).

[50] See https://github.com/pauxy-qmc/pauxy for details on how to obtain the source code.

[51] P. R. C. Kent, Abdulgani Annaberdiyev, Anouar Benali, M. Chandler Bennett, Edgar Josué Landinez Borda, Peter Doak, Hongxia Hao, Kenneth D. Jordan, Jaron T. Krogel, Ilkka Kylänpää, Joonho Lee, Ye Luo, Fionn D. Malone, Cody A. Melton, Lubos Mitas, Miguel A. Morales, Eric Neuscamman, Fernando A. Reboredo, Brenda Rubenstein, Kayahan Saritas, Shiv Upadhyay, Guangming Wang, Shuai Zhang, and Luning Zhao, "QMCPACK: Advances in the development, efficiency, and application of auxiliary field and real-space variational and diffusion quantum Monte Carlo," J. Chem. Phys. **152**, 174105 (2020).

[52] Qiming Sun, Timothy C. Berkelbach, Nick S. Blunt, George H. Booth, Sheng Guo, Zhendong Li, Junzi Liu, James D. McClain, Elvira R. Sayfutyarova, Sandeep Sharma, Sebastian Wouters, and Garnet Kin Lic Chan, "Pyscf: the python-based simulations of chemistry framework," WIREs Comput. Mol. Sci. **8**, e1340 (2017).

[53] Evgeny Epifanovsky, Andrew T. B. Gilbert, Xintian Feng, Joonho Lee, Yuezhi Mao, Narbe Mardirossian, Pavel Pokhilko, Alec F. White, Marc P. Coons, Adrian L. Dempwolff, Zhengting Gan, Diptarka Hait, Paul R. Horn, Leif D. Jacobson, Ilya Kaliman, Jörg Kussmann, Adrian W. Lange, Ka Un Lao, Daniel S. Levine, Jie Liu, Simon C. McKenzie, Adrian F. Morrison, Kaushik D. Nanda, Felix Plasser, Dirk R. Rehn, Marta L. Vidal, Zhi-Qiang You, Ying Zhu, Bushra Alam, Benjamin J. Albrecht, Abdulrahman Aldossary, Ethan Alguire, Josefine H. Andersen, Vishikh Athavale, Dennis Barton, Khadiza Begam, Andrew Behn, Nicole Bellonzi, Yves A. Bernard, Eric J. Berquist, Hugh G. A. Burton, Abel Carreras, Kevin Carter-Fenk, Romit Chakraborty, Alan D. Chien, Kristina D. Closser, Vale Cofer-Shabica, Saswata Dasgupta, Marc de Wergifosse, Jia Deng, Michael Diedenhofen, Hainam Do, Sebastian Ehlert, Po-Tung Fang, Shervin Fatehi, Qingguo Feng, Triet Friedhoff, James Gayvert, Qinghui Ge, Gergely Gidofalvi, Matthew Goldey, Joe Gomes, Cristina E. González-Espinoza, Sahil Gulania, Anastasia O. Gunina, Magnus W. D. Hanson-Heine, Phillip H. P. Harbach, Andreas Hauser, Michael F. Herbst, Mario Hernández Vera, Manuel Hodecker, Zachary C. Holden, Shannon Houck, Xunkun Huang, Kerwin Hui, Bang C. Huynh, Maxim Ivanov, Ádám Jász, Hyunjun Ji, Hanjie Jiang, Benjamin Kaduk, Sven Kähler, Kirill Khistyaev, Jaehoon Kim, Gergely Kis, Phil Klunzinger, Zsuzsanna Koczor-Benda, Joong Hoon Koh, Dimitri Kosenkov, Laura Koulias, Tim Kowalczyk, Caroline M. Krauter, Karl Kue, Alexander Kunitsa, Thomas Kus, István Ladjánszki, Arie Landau, Keith V. Lawler, Daniel Lefrancois, Susi Lehtola, Run R. Li, Yi-Pei Li, Jiashu Liang, Marcus Liebenthal, Hung-Hsuan Lin, You-Sheng Lin, Fenglai Liu, Kuan-Yu Liu, Matthias Loipersberger, Arne Luenser, Aaditya Manjanath, Prashant Manohar, Erum Mansoor, Sam F. Manzer, Shan-Ping Mao, Aleksandr V. Marenich, Thomas Markovich, Stephen Mason, Simon A. Maurer, Peter F. McLaughlin, Maximilian F. S. J. Menger, Jan-Michael Mewes, Stefanie A. Mewes, Pierpaolo Morgante, J. Wayne Mullinax, Katherine J. Oosterbaan, Garrette Paran, Alexander C. Paul, Suranjan K. Paul, Fabijan Pavošević, Zheng Pei, Stefan Prager, Emil I. Proynov, Ádám Rák, Eloy Ramos-Cordoba, Bhaskar Rana, Alan E. Rask, Adam Rettig, Ryan M. Richard, Fazle Rob, Elliot Rossomme, Tarek Scheele, Maximilian Scheurer, Matthias Schneider, Nickolai Sergueev, Shaama M. Sharada, Wojciech Skomorowski, David W. Small, Christopher J. Stein, Yu-Chuan Su, Eric J. Sundstrom, Zhen Tao, Jonathan Thirman, Gábor J. Tornai, Takashi Tsuchimochi, Norm M. Tubman, Srimukh Prasad Veccham, Oleg Vydrov, Jan Wenzel, Jon Witte, Atsushi Yamada, Kun Yao, Sina Yeganeh, Shane R. Yost, Alexander Zech, Igor Ying Zhang, Xing Zhang, Yu Zhang, Dmitry Zuev, Alán Aspuru-Guzik, Alexis T. Bell, Nicholas A. Besley, Ksenia B. Bravaya, Bernard R. Brooks, David Casanova, Jeng-Da Chai, Sonia Coriani, Christopher J. Cramer, György Cserey, A. Eugene

DePrince, Robert A. DiStasio, Andreas Dreuw, Barry D. Dunietz, Thomas R. Furlani, William A. Goddard, Sharon Hammes-Schiffer, Teresa Head-Gordon, Warren J. Hehre, Chao-Ping Hsu, Thomas-C. Jagau, Yousung Jung, Andreas Klamt, Jing Kong, Daniel S. Lambrecht, WanZhen Liang, Nicholas J. Mayhall, C. William McCurdy, Jeffrey B. Neaton, Christian Ochsenfeld, John A. Parkhill, Roberto Peverati, Vitaly A. Rassolov, Yihan Shao, Lyudmila V. Slipchenko, Tim Stauch, Ryan P. Steele, Joseph E. Subotnik, Alex J. W. Thom, Alexandre Tkatchenko, Donald G. Truhlar, Troy Van Voorhis, Tomasz A. Wesolowski, K. Birgitta Whaley, H. Lee Woodcock, Paul M. Zimmerman, Shirin Faraji, Peter M. W. Gill, Martin Head-Gordon, John M. Herbert, and Anna I. Krylov, "Software for the frontiers of quantum chemistry: An overview of developments in the Q-Chem 5 package," J. Chem. Phys. **155**, 084801 (2021).

[54] Adam A. Holmes, Norm M. Tubman, and C. J. Umrigar, "Heat-Bath Configuration Interaction: An Efficient Selected Configuration Interaction Algorithm Inspired by Heat-Bath Sampling," J. Chem. Theory Comput. **12**, 3674–3680 (2016).

[55] Joonho Lee and Martin Head-Gordon, "Regularized Orbital-Optimized Second-Order Møller–Plesset Perturbation Theory: A Reliable Fifth-Order-Scaling Electron Correlation Model with Orbital Energy Dependent Regularizers," J. Chem. Theory Comput. **14**, 5203–5219 (2018).

[56] Thom H. Dunning, "Gaussian basis sets for use in correlated molecular calculations. I. The atoms boron through neon and hydrogen," J. Chem. Phys. **90**, 1007–1023 (1989).

[57] S. Goedecker, M. Teter, and J. Hutter, "Separable dual-space Gaussian pseudopotentials," Phys. Rev. B **54**, 1703–1710 (1996).

[58] Joost VandeVondele and Jürg Hutter, "Gaussian basis sets for accurate calculations on molecular systems in gas and condensed phases," J. Chem. Phys. **127**, 114105 (2007).

[59] Abhinav Kandala, Antonio Mezzacapo, Kristan Temme, Maika Takita, Markus Brink, Jerry M Chow, and Jay M Gambetta, "Hardware-efficient variational quantum eigensolver for small molecules and quantum magnets," Nature **549**, 242–246 (2017).

[60] Yunseong Nam, Jwo-Sy Chen, Neal C Pisenti, Kenneth Wright, Conor Delaney, Dmitri Maslov, Kenneth R Brown, Stewart Allen, Jason M Amini, Joel Apisdorf, *et al.*, "Ground-state energy estimation of the water molecule on a trapped-ion quantum computer," npj Quantum Information **6**, 1–6 (2020).

[61] Google AI Quantum *et al.*, "Hartree-fock on a superconducting qubit quantum computer," Science **369**, 1084–1089 (2020).

[62] Frank Arute, Kunal Arya, Ryan Babbush, Dave Bacon, Joseph C Bardin, Rami Barends, Andreas Bengtsson, Sergio Boixo, Michael Broughton, Bob B Buckley, *et al.*, "Observation of separated dynamics of charge and spin in the fermi-hubbard model," arXiv preprint arXiv:2010.07965 (2020).

[63] Dmitri Maslov and Martin Roetteler, "Shorter stabilizer circuits via bruhat decomposition and quantum circuit transformations," IEEE Transactions on Information Theory **64**, 4729–4738 (2018).

[64] Fionn D. Malone, Shuai Zhang, and Miguel A. Morales, "Overcoming the memory bottleneck in auxiliary field quantum monte carlo simulations with interpolative separable density fitting," J. Chem. Theory. Comput. **15**, 256 (2019).

[65] Norbert Schuch and Frank Verstraete, "Computational complexity of interacting electrons and fundamental limitations of density functional theory," Nature Physics **5**, 732–735 (2009).

[66] Daniel S Abrams and Seth Lloyd, "Quantum Algorithm Providing Exponential Speed Increase for Finding Eigenvalues and Eigenvectors," Physical Review Letters **83**, 5162–5165 (1999).

[67] Sandro Sorella, "Linearized auxiliary fields Monte Carlo technique: Efficient sampling of the fermion sign," Phys. Rev. B **84**, 241110 (2011).

[68] Ankit Mahajan and Sandeep Sharma, "Taming the Sign Problem in Auxiliary-Field Quantum Monte Carlo Using Accurate Wave Functions," J. Chem. Theory Comput. **17**, 4786–4798 (2021).
